# Enhanced Bottom-Up and Reduced Top-Down fMRI Activity Is Related to Long-Lasting Nonreinforced Behavioral Change

**DOI:** 10.1093/cercor/bhz132

**Published:** 2019-08-13

**Authors:** Rotem Botvinik-Nezer, Tom Salomon, Tom Schonberg

**Affiliations:** 1 Sagol School of Neuroscience, Tel Aviv University, Tel Aviv 6997801, Israel; 2 Faculty of Life Sciences, Department of Neurobiology, Tel Aviv University, Tel Aviv 6997801, Israel

**Keywords:** behavioral change, decision making, fMRI, preferences, value

## Abstract

Behavioral change studies and interventions focus on self-control and external reinforcements to influence preferences. Cue-approach training (CAT) has been shown to induce preference changes lasting months by merely associating items with neutral cues and speeded responses. We utilized this paradigm to study neural representation of preferences and their modification without external reinforcements. We scanned 36 participants with fMRI during a novel passive viewing task before, after and 30 days following CAT. We preregistered the predictions that activity in memory, top-down attention, and value-processing regions will underlie preference modification. While most theories associate preferences with prefrontal regions, we found that “bottom-up” perceptual mechanisms were associated with immediate change, whereas reduced “top-down” parietal activity was related to long-term change. Activity in value-related prefrontal regions was enhanced immediately after CAT for trained items and 1 month after for all items. Our findings suggest a novel neural mechanism of preference representation and modification. We suggest that nonreinforced change of preferences occurs initially in perceptual representation of items, putatively leading to long-term changes in “top-down” processes. These findings offer implementation of bottom-up instead of top-down targeted interventions for long-lasting behavioral change.

## Introduction

Changing behavior is key to solving a broad range of challenges in public health. Understanding how preferences are constructed and modified is a major challenge in the research of human behavior with broad implications, from basic science to offering long-lasting behavioral change programs (Vlaev et al. 2011; Marteau et al. 2012). Most behavioral interventions for treating conditions such as addictions and eating disorders currently rely on reinforcements and effortful self-control (Wood and Neal 2016). However, previous studies suggest that these interventions tend to fail in the long term (Jeffery et al. 2000; Prochaska et al. 2004; Christiansen et al. 2007).

In a recently introduced paradigm, named cue-approach training (CAT), preferences for snack food items were successfully modified in the absence of external reinforcements (Schonberg et al. 2014). In the CAT paradigm, the mere association of images of items with a cue and a speeded button-press response lead to preference changes lasting months following a single training session (Schonberg et al. 2014; Salomon et al. 2018). Current theories in the field of value-based decision-making would not predict that a simple association of an image with a neutral cue and a button press will affect choices lasting months into the future. However, replicated results of over 30 samples in multiple laboratories show that participants significantly choose high-value paired items (“Go items”) over high-value nonpaired items (“NoGo items”) following CAT (Schonberg et al. 2014; Bakkour et al. 2016, 2017, 2018; Veling et al. 2017; Zoltak et al. 2017). Salomon et al. (2018) recently showed that CAT can be used to change preferences toward various types of stimuli (snack food items, unfamiliar faces, fractal art images, and positive affective images) with different types of cues (neutral auditory, aversive auditory, and visual cues), demonstrating that the underlying mechanisms of the effect are general. Previous studies showed that the motor response (Schonberg et al. 2014) and the cue onset and its challenging nature (Bakkour et al. 2016) are crucial for the CAT effect. Omission of any one of these components diminished the preference modification effect following CAT. Other experiments found that CAT also affects preferences toward healthy food items (Veling et al. 2017) and can be induced when choices are made with the eyes rather than the hands (Bakkour et al. 2016).

Preference change following the task has been shown to last up to 6 months following a single training session of less than 1 h (Schonberg et al. 2014; Salomon et al. 2018), suggesting that the task has potential to be translated into a real-world intervention and that it involves long-term memory components.

Training in the task is performed on single items and thus induces changes of preferences toward individual items, later manifested in the binary choice phase. The low-level nature of the task, involving neither external reinforcements nor high-level executive control, but rather sensory-motor associations, provides a unique opportunity to study in relative isolation preference representation and modification in the brain.

The underlying neural mechanisms driving this replicable long-lasting change remain largely unknown. Previous studies showed that eye gaze during binary choices, following CAT, was drawn toward high-value Go items more compared with high-value NoGo items, even when the Go items were not chosen (Schonberg et al. 2014). Functional MRI (fMRI), during choices of high-value Go items alone and compared with choices of high-value NoGo items, demonstrated an amplified BOLD signal in the ventro-medial prefrontal cortex (vmPFC) (Schonberg et al. 2014), a region associated with value-based decision-making (Chib et al. 2009). Together, these results indicate the involvement of attentional mechanisms and a neural signature of the value change during choices of Go compared with choices of NoGo items. Overall, these previous studies demonstrated that fMRI data during training and choices were not sufficient to reveal the underlying neural mechanisms of preference change induced by the task (via training of individual items in the absence of external reinforcements).

Therefore, here we aimed to study how preferences toward individual items are changed in the task and uncover how individual items’ value is represented and modified in the brain even without external reinforcements. To do so, we introduce a novel passive viewing task, whereby pictures of snack food items are individually presented on the screen, while participants perform a sham counting task. This task was performed and scanned before, after, and 1 month following CAT. By comparing fMRI activity during this task, we aimed to test the different neural responses to the same images of Go versus NoGo items after training compared with baseline, as well as for the first time the neural changes 1 month following training. Regions in the brain showing preference-related functional plasticity immediately after training and 1 month later could reveal a novel mechanism of preference representation in the brain and specifically indicate how nonexternally reinforced training leads to robust long-lasting preference changes.

We hypothesized that preference changes are dependent on attentional and memory-related mechanisms, affecting value representation. Based on previous findings (Schonberg et al. 2014; Veling et al. 2017), we hypothesized that attention-related processes are involved in the behavioral change following CAT and focused our predictions on top-down attention-related regions. Moreover, we hypothesized that memory processes are involved in the neural mechanism underlying the behavioral change following CAT, in the short and long term. This is following the findings that the preference changes induced by the task, lasted for months after a single training session (Schonberg et al. 2014; Salomon et al. 2018) and based on recent theories for the involvement of memory in value-based decision-making (Weber and Johnson 2006; Wimmer and Shohamy 2012; Shohamy and Daw 2015; Shadlen and Shohamy 2016). Finally, as was previously demonstrated during the choice phase following CAT (Schonberg et al. 2014; Bakkour et al. 2017), we hypothesized we will observe neural changes in pre-frontal value-related regions. In our preregistered hypotheses we predicted greater BOLD activity after CAT in response to high-value Go items in episodic memory-related regions in the medial temporal lobe (Brown et al. 2015), top-down attention-related dorsal parietal cortex (Corbetta and Shulman 2002; Cabeza et al. 2008), and prefrontal value-related regions (Kable and Glimcher 2009; Padoa-Schioppa 2011). In addition, we hypothesized that we will replicate previous CAT results showing a significant behavioral effect of choosing high-value Go over high-value NoGo items during the binary choice probe phase, and enhanced BOLD activity in the vmPFC during choices of high-value Go items (Schonberg et al. 2014; Bakkour et al. 2016, 2017, 2018; Veling et al. 2017; Zoltak et al. 2017; Salomon et al. 2018).

Understanding the neural mechanisms underlying nonreinforced behavioral change could potentially set the ground for new theories of value-based decision-making, and for new behavioral change interventions targeting automatic processes for long-lasting change, benefiting the lives of millions around the world.

## Materials and Methods

### Data Sharing

We preregistered our sample size, hypotheses, and a “general” analysis plan (prior to final full analyses) on the Open Science Framework (OSF; project page, https://osf.io/x6hsq/ preregistration, https://osf.io/yu3tw/). Deviations from the preregistered general analysis plan are described below. The behavioral data and analysis codes are also available on the preregistered OSF project. Imaging data are available in Brain Imaging Data Structure (BIDS) format (Gorgolewski et al. 2016) on OpenNeuro (as well as FSL design.fsf files, confounds.tsv files, and the regions of interest’s masks): https://openneuro.org/datasets/ds001417. Unthresholded and thresholded statistical images of the imaging results are available on NeuroVault (Gorgolewski et al. 2015): https://neurovault.org/collections/TTZTGQNU/.

### Participants

Forty healthy right-handed participants took part in this experiment. The sample size was chosen before data collection and preregistered during data collection. We initially planned to collect *n* = 35 participants based on a power analysis using a previous imaging CAT sample (Schonberg et al. 2014), aimed to detect with 80% power and α = 0.05 enhanced parametric modulation of activity in the vmPFC during probe choices of high-value Go over high-value NoGo items. During data collection, we found that attrition rates were higher than expected, thus the planned sample size was increased to *n* = 40 (before exclusions and attrition) and re-registered. The total number of valid participants included in the final analyses of the first session is *n* = 36 (19 females; age: mean = 26.11, SD = 3.46 years). Of these participants, *n* = 27 returned for an additional 1-month follow-up session (15 females; age: mean = 26.15, SD = 3.44 years). Due to a scanner upgrade, we were unable to complete the entire follow-up cohort as planned.

All participants had normal or corrected-to-normal vision and hearing; had no history of eating disorders or psychiatric, neurologic or metabolic diagnoses; had no food restrictions; and were not taking any medications that would interfere with the experiment. Participants were asked to refrain from eating for 4 h prior to arrival to the laboratory (Schonberg et al. 2014). All participants gave informed consent. The study was approved by the institutional review board at the Sheba Tel Hashomer Medical Center and the ethics committee at Tel Aviv University.

#### Exclusions

A total of 4 out of the 40 participants were excluded: one participant due to incompletion of the experiment, one based on training exclusion criteria (7.5% false alarm rate during training), and 2 participants with incidental brain findings; resulting in *n* = 36 valid participants.

### Stimuli

Sixty color images of familiar local snack food items were used in the current experiment. Images depicted the snack package and the snack itself on a homogenous black rectangle sized 576 × 432 pixels (see Supplementary Table 1; stimuli dataset was created in our lab and is available online at http://schonberglab.tau.ac.il/resources/snack-food-image-database/). All snack food items were also available for actual consumption at the end of the experiment. Participants were presented with the real food items at the beginning of the experiment in order to promote incentive compatible behavior throughout the following tasks.

### Experimental Procedures

**Figure 1:**
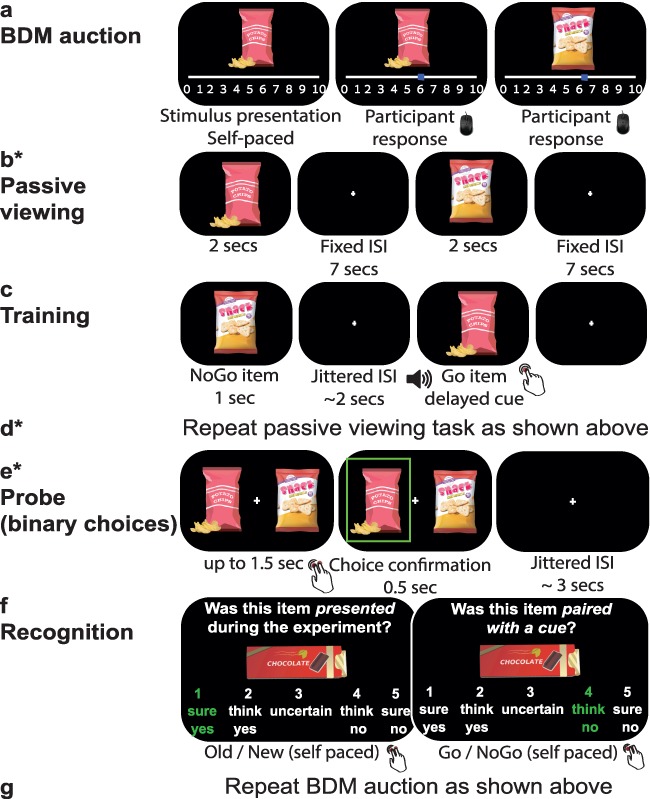
Outline of the experimental procedures: procedures performed inside the MRI scanner are marked with an asterisk. (*a*) Initial preferences were evaluated using the BDM auction procedure. (*b*) In the passive viewing task, items were individually presented on the screen. (*c*) CAT: Participants were instructed to press a button as fast as they could whenever they heard an auditory cue. Go items were consistently paired with the cue and button press response, while NoGo items were not. (*d*) The passive viewing task was repeated after training. (*e*) In the probe task, participants chose their preferred item between pairs of items with similar initial subjective preferences, one Go and one NoGo item. (*f*) A recognition memory task. (*g*) The BDM auction was repeated. Tasks d–g were performed again in the 1-month follow-up session.

The general task procedure was similar to previous studies with CAT (Schonberg et al. 2014; Salomon et al. 2018) and is presented in Fig. 1. In order to test for functional changes in the neural response to the individual items following CAT, we added a new passive viewing task before, after, and 1 month following training.

First, we obtained the subjective willingness to pay (WTP) of each participant for each of the 60 snack food items using the Becker–DeGroot–Marschak (BDM) auction procedure (Becker et al. 1964), performed outside the MRI scanner (see Fig. 1*a,g*). Then, participants entered the scanner and completed 2 passive viewing runs while scanned with fMRI (see Fig. 1*b,d*), followed by anatomical and diffusion-weighted imaging (DWI) scans. Afterwards, participants went out of the scanner and completed CAT in a behavioral testing room at the imaging center (see Fig. 1*c*). They then returned to the scanner and were scanned again with anatomical and DWI. Then, they were scanned with fMRI while performing 2 more runs of the passive viewing task and 4 runs of the probe phase, during which they chose between pairs of items (see Fig. 1*e*). Finally, participants completed a recognition task outside the scanner (see Fig. 1*f*). As the last task of the first session, they again completed the BDM auction to obtain their WTP for the snacks.

Approximately 1 month after the first session of the experiment, participants returned to the lab. They entered the scanner, were scanned with anatomical and DWI scans, and completed 2 passive viewing runs as well as another probe phase (without additional training). Finally, participants completed the recognition and BDM auction parts, outside the scanner.

Anatomical and DWI data were obtained for each participant before, immediately after, and 1 month following training, as well as from a control group, used for the diffusion MRI part of the experiment. Analyses of diffusion data are not included in this paper.

#### Initial Preferences Evaluation (see Fig. 1*a,g*)

In order to obtain initial subjective preferences, participants completed the BDM auction procedure (Becker et al. 1964). Participants first received 10 Israeli Shekels (ILS; equivalent to approx. US$2.7). During the auction, 60 snack food items were presented on the screen one after the other in random order. For each item, participants were asked to indicate their WTP for the presented item. Participants placed their bid for each item using the mouse cursor along a visual analog scale, ranging from 0 to 10 ILS with 1/450 ILS increments (task was self-paced). Participants were told in advance that at the end of the experiment, the computer will randomly generate a counter bid ranging from 0 to 10 ILS (with 0.5 increments) for 1 of the 60 items. If the bid placed by the participant exceeds the computer’s bid, he or she will be required to buy the item for the computer’s lower bid price. Otherwise, the participant will not be allowed to buy the snack but gets to retain the allocated 10 ILS. Participants were told that, at the end of the experiment, they will stay in the room for 30 min and the only food they will be allowed to eat is the snack (in case they “won” the auction and purchased it). Participants were explicitly instructed that the best strategy for this task was to indicate their actual WTP for each item.

The auction task was completed twice in the first session of the experiment: once at the beginning and once at the end of the session. During the second auction, participants were instructed that at the end of the experiment a single trial will be randomly chosen from either the first or the second auction, to be actualized.

#### Item Selection

For each participant, items were rank ordered from 1 (highest value) to 60 (lowest value) based on their WTP. Then, 12 items (ranked 7–18) were defined as high-value items to be used in probe, and 12 items (ranked 43–54) were defined as low-value items to be used in probe. Each group of 12 items (high-value or low-value) was split to 2 subgroups with identical mean rank. Six of the 12 items were chosen to be paired with the cue during training (Go items; training procedures are described in the following sections), and the other 6 were not paired with the cue during training (NoGo items). This allowed us to pair high-value Go and high-value NoGo items, or low-value Go and low-value NoGo items, with similar initial WTPs, for the probe binary choices. To maintain 30% frequency of Go items during training, similar to previous studies with CAT (Schonberg et al. 2014; Bakkour et al. 2016, 2017; Salomon et al. 2018), we used 16 additional NoGo items. These items were also used during training and passive viewing (40 snacks overall; see Supplementary Fig. 1 and Supplementary Table 2 for a detailed description of all stimuli allocation).

#### Passive Viewing (see Fig. 1*b,d*)

The task was performed inside the scanner, while participants were scanned with fMRI. This new task was introduced to evaluate the functional changes in the neural response to the individual items following CAT. The neural signature of the participants’ response to each of the individual items was obtained in 3 different time points: a baseline measurement before CAT, after CAT, and in a 1-month follow-up. In this task, participants passively viewed a subset of 40 items, which were also presented during training (see item selection section and Supplementary Fig. 1). The task consisted of 2 runs (in each session). On each run, each of the 40 items was presented on the screen for a fixed duration of 2 s, followed by a fixed inter-stimulus interval (ISI) of 7 s. Items were presented in random order. To ensure participants were observing and processing the presented images, we asked them to perform a sham task of silently counting how many items were of snacks containing in a new package either one piece (e.g., a “Mars” chocolate bar) or several pieces (e.g., “M&M's”). At the end of each run, participants were asked how many items they counted. Task instructions (count one/several) were counterbalanced between runs for each participant. The time elapsed between the 2 runs before training and 2 runs after training was about 2 h (including CAT, anatomical and diffusion weighted scans before and after training, and time to exit and enter the scanner).

#### Cue-Approach Training (see Fig. 1*c*)

Training was performed outside the scanner. The training task included the same 40 items presented in the passive viewing task. Each image was presented on the screen one at a time for a fixed duration of 1 s. Participants were instructed to press a button on the keyboard as fast as they could when they heard an auditory cue, which was consistently paired with 30% of the items (Go items). Participants were not informed in advance that some of the items consistently paired with the cue, or the identity of the Go items. The auditory cue consisted of a 180-ms-long sinus wave function. The auditory cue was heard initially 750 ms after stimulus onset (Go-signal delay, GSD). To ensure a success rate of around 75% in pressing the button before stimulus offset, we used a ladder technique to update the GSD. The GSD was increased by 16.67 ms following every successful trial and decreased by 50 ms if the participant did not press the button or pressed it after the offset of the stimulus (1:3 ratio). Items were followed by a fixation cross that appeared on the screen for a jittered ISI with an average duration of 2 s (range: 1–6 s). Each participant completed 20 repetitions of training; each repetition included all 40 items presented in random order. A short break was given following every 2 training repetitions, in which the participants were asked to press a button when they were ready to proceed. The entire training session lasted about 40 to 45 min, depending on the duration of the breaks, which were controlled by the participants.

#### Probe (see Fig. 1*e*)

The probe was conducted while participants were scanned with fMRI. The probe phase was aimed to test participants’ preferences following training and as in previous studies (Schonberg et al. 2014; Bakkour et al. 2016, 2017; Veling et al. 2017; Zoltak et al. 2017; Salomon et al. 2018) was the central behavioral measure of the effectiveness of CAT. Participants were presented with pairs of items that had similar initial rankings (high-value or low-value), but only one of the items in each pair was associated with the cue during training (e.g., high-value Go vs. high-value NoGo). They were given 1.5 s to choose the item they preferred on each trial, by pressing one of 2 buttons on an MRI-compatible response box. Their choice was highlighted for 0.5 s with a green rectangle around the chosen items. If they did not respond on time, a message appeared on the screen, asking them to respond faster. A fixation cross appeared at the center of the screen between the 2 items during each trial, as well as during the ISI, which lasted on average 3 s (range, 1–12 s).

The probe phase consisted of 2 blocks. On each block, each of the 6 high-value Go items were compared with each of the 6 high-value NoGo items (36 comparisons), as well as each of the 6 low-value Go items with each of the 6 low-value NoGo items. Thus, overall there were 72 pairs of Go–NoGo comparisons (each repeated twice during probe, once on each block). In addition, on each block we compared 2 high-value NoGo items with 2 low-value NoGo items, resulting in 4 probe pairs that were used as “sanity checks” to ensure participants chose the items they preferred according to the initial WTP values obtained during the BDM auction. Each probe block was divided to 2 runs, each consisted of half of the total 76 unique pairs (38 trials on each run). All pairs within each run were presented in random order, and the location of the items (left/right) was also randomly chosen. Choices during the probe phase were made for consumption to ensure they were incentive compatible. Participants were told that a single trial will be randomly chosen at the end of the experiment and that they will receive the item they chose on that specific trial. At the time of the BDM auctions, participants still did not know which binary choice will be chosen from the probe phase and thus the auction procedure should still have been valid at eliciting their WTP. The participants were shown the snack box with all snacks prior to the beginning of the experiment.

#### Recognition Task (see Fig. 1*f*)

Participants completed a recognition task, outside the scanner. In this task, the items from the probe phase, as well as an equal number of new items, were presented on the screen one by one and participants were asked to indicate for each item whether or not it was presented during the experiment and whether or not it was paired with the cue during training. The first 5 participants completed a binary version of the recognition task: they first completed the old/new recognition task for all the items and were then presented again with all the items they recognized as old items and were asked whether or not each item was paired with the cue (Go/NoGo recognition task). In the follow-up session, they were again presented with the items they indicated were old items (in the first session), in random order, and were asked again whether each item was paired with the auditory cue or not. In this version, each response was a binary yes/no response (“Was this item presented during the experiment?”). The rest of the participants completed a different version of the task. For each answer, they had 5 possible responses: certain yes, think yes, uncertain, think no, or certain no. The items were presented one by one on the screen, and for each item the participant was first asked whether this item was presented during the experiment and then, independent of the response to the first question, was asked whether or not it was paired with a cue during training.

#### One-Month Follow-up Session

All participants were invited to the follow-up session approximately 1 month after training. A subset of 27 participants returned to the lab and completed the follow-up session. They were scanned with anatomical and diffusion protocols, completed 2 passive viewing runs, and performed another probe while scanned with fMRI protocols, similar to the first session. In the follow-up session, the probe included the same pairs as the probe of the first session, presented in a new random order. Afterwards, participants completed another session of the recognition task and a third BDM auction, both outside the scanner in the testing room.

### Behavioral Analysis

Analyses that were preregistered with specific directional predictions were performed using one-sided statistical tests. The participants were included in all models as a random effect. Exploratory analyses that were not preregistered can be found in the Supplementary Material (Supplementary exploratory analyses section).

#### Probe

As the central behavioral measure of the CAT effect, similar to previous studies using cue-approach task (Schonberg et al. 2014; Salomon et al. 2018), we performed a repeated-measures logistic regression to compare the odds of choosing Go items against chance level (log odds = 0; odds ratio = 1) for each trial type (high-value/low-value). We also compared the ratio of choosing the Go items between high-value and low-value pairs. These analyses were conducted for each session separately.

In addition, we performed exploratory analyses of the probe data. We tested the correlation between choices in the first session with choices in the follow-up session, the effect of CAT on choices above and beyond the baseline WTP difference between the 2 items in each probe pair and the response times (RTs) of choices (see Supplementary exploratory analyses: correlation between choices across sessions, the effect of CAT on choices above and beyond baseline WTP difference, choices RTs).

#### Recognition memory

In order to similarly analyze the recognition data across the 2 versions of the task, responses from the second version (i.e., with the confidence levels) were converted to binary yes/no answers. “Uncertain” was considered a wrong answer. In order to test whether Go items were better remembered than NoGo items following CAT, we compared the hit rate (the percent of old items that were correctly recognized as such) as well as RT in the old/new recognition task between Go and NoGo items that were included in the Go versus NoGo probe pairs (i.e., 6 high-value Go items, 6 high-value NoGo items, 6 low-value Go items, and 6 low-value NoGo items). For the RT analysis, we only included trials with correct responses, under the assumption that shorter RTs for correct responses reflect better memory, while shorter RTs for incorrect responses do not.

It should be noted that this task was completed immediately after the probe task, both in the first and in the follow-up session. Hence, in the follow-up session the old/new task again tested memory for the same session, rather than long-term memory of the first session. Therefore, the Go/NoGo recognition task may be a better indication for long-term memory, but for the associations created during training (between the cue and Go items) rather than for the items themselves. Moreover, in the binary version of the task during the follow-up session (the first 4 participants in the follow-up session), only the Go/NoGo recognition task was performed. In addition, the recognition task (both versions and sessions) was self-paced, and thus the RT measure included outliers, for example when participants took a break. Therefore, trials with RT longer than 3 standard deviations (SDs) above the mean across all trials of all participants for each version of the task were excluded from analysis.

We tested whether participants significantly remembered the cue-item associations separately for each session, with a logistic regression model comparing the odds of answering correctly to the Go/NoGo recognition task against 50% chance level (log odds = 0; odds ratio = 1). We also tested the linear correlation between the accuracy in the Go/NoGo recognition task (hits and correct rejections, only for items that are included in the Go/NoGo probe choices) and the proportion of choosing Go over NoGo items across participants. As in the analysis of the old/new recognition task, we only included the 24 items that comprised the Go/NoGo probe comparisons, to control for the number of times participants viewed each item as well as for the ratio of Go versus NoGo items (which was therefore 1:1 in the recognition analyses).

#### Auction

Similarly to the original CAT study (Schonberg et al. 2014), we tested if WTP changed over time differently for Go versus NoGo items. Due to the fact that this measure was not always been replicable in previous studies, it was not the main measure of effectiveness of CAT in the current study. We computed ΔWTP (WTP after minus WTP before) for each item and each participant. Then, we used a repeated-measures linear mixed model with the ΔWTP as dependent variable and item type (Go/NoGo) and WTP before as independent variables. We were interested in the main effect of item type, that is, whether ΔWTP was different for Go versus NoGo items. This analysis was performed separately for high-value and low-value items, as well as for the short-term change (after CAT minus before CAT) and the long-term change (1 month following CAT minus before CAT). In addition, we performed exploratory analyses to test whether there was a choice effect on WTP (see Supplementary exploratory analyses: choice effect on WTP).

#### Training

We performed exploratory analyses to test whether responses during training were related to choices following training (see Supplementary exploratory analyses: training response times and choices).

### MRI Data Acquisition

Imaging data were acquired using a 3 T Siemens Prisma MRI scanner with a 64-channel head coil, at the Strauss imaging center on the campus of Tel Aviv University. Functional data were acquired using a T2*-weighted echo planar imaging sequence. Repetition time (TR) = 2000 ms, echo time (TE) = 30 ms, flip angle (FA) = 90 degrees, field of view (FOV) = 224 × 224 mm, acquisition matrix of 112 × 112. We positioned 58 oblique axial slices with a 2 × 2 mm in plane resolution 15 degrees off the anterior commissure posterior commissure line to reduce the frontal signal dropout (Deichmann et al. 2003), with a space of 2 mm and a gap of 0.5 mm to cover the entire brain. We used a multiband sequence (Moeller et al. 2010) with acceleration factor = 2 and parallel imaging factor (iPAT) = 2, in an interleaved fashion. Each of the passive viewing runs consisted of 180 volumes and each of the probe runs consisted of 100 volumes. In addition, in each scanning session (before, after, and 1 month following training) we acquired high-resolution T1w structural images using a magnetization prepared rapid gradient echo (MPRAGE) pulse sequence (TR = 1.75 s, TE = 2.59 ms, FA = 8°, FOV = 224 × 224 × 208 mm, resolution = 1 × 1 × 1 mm for the first 5 participants; TR = 2.53 s, TE = 2.88 ms, FA = 7°, FOV = 224 × 224 × 208 mm, resolution = 1 × 1 × 1 mm for the rest of the sample. Protocol was changed to enhance the T1w contrast and improve registration of the functional data to the standard space).

### fMRI Preprocessing

Raw imaging data in DICOM format were converted to NIfTI format with dcm2nii tool (Li et al. 2016). The NIfTI files were organized according to the BIDS format v1.0.1 (Gorgolewski et al. 2016). These data are publicly shared on OpenNeuro (https://openneuro.org/datasets/ds001417). Preprocessing of the functional imaging data was performed using fMRIprep version 1.0.0-rc8 (Esteban et al. 2019), a Nipype based tool. Each T1-weighted volume was corrected for bias field using N4BiasFieldCorrection v2.1.0 and skull stripped using antsBrainExtraction.sh v2.1.0 (using OASIS template). Cortical surface was estimated using FreeSurfer v6.0.0 (Dale et al. 1999). The skull-stripped T1-weighted volumes were co-registered to skull stripped ICBM 152 Nonlinear template version 2009c (Fonov et al. 2009) using nonlinear transformation implemented in ANTs v2.1.0 (Avants et al. 2008). Functional data were motion corrected using MCFLIRT v5.0.9. This was followed by coregistration to the corresponding T1-weighted volume using boundary-based registration with 9 degrees of freedom, implemented in FreeSurfer v6.0.0. Motion correcting transformations, T1-weighted transformation, and MNI template warp were applied in a single step using antsApplyTransformations v2.1.0 with Lanczos interpolation. Three tissue classes were extracted from the T1-weighted images using FSL FAST v5.0.9. Voxels from cerebrospinal fluid and white matter were used to create a mask in turn used to extract physiological noise regressors using aCompCor. Mask was eroded and limited to subcortical regions to limit overlap with gray matter, and 6 principal components were estimated. Framewise displacements were calculated for each functional run using Nipype implementation. For more details of the pipeline using fMRIprep see http://fmriprep.readthedocs.io/en/1.0.0/workflows.html.

We created confound files for each scan (each run of each task of each session of each participant), with the following measurements: SD of the root mean squared intensity difference from one volume to the next (DVARS), absolute DVARS values, voxelwise SD of DVARS values, and 6 motion parameters (translational and rotation, each in 3 directions). We added a single time point regressor (a single additional column) for each volume with framewise-displacement value larger than 0.9, in order to model out volumes with extensive motion (i.e., scrubbing). Scans with more than 15% scrubbed volumes were excluded from analysis, resulting in one excluded participant from the analysis of the first session’s probe task. The confounds.tsv files (FSL format) can be found with the data shared on OpenNeuro.

### fMRI Analysis

Imaging analysis was performed using FEAT (fMRI Expert Analysis Tool) v6.00, part of FSL (FMRIB Software Library) (Smith et al. 2004).

#### Univariate Imaging Analysis—Passive Viewing

The functional data from the passive viewing task were used to examine the functional changes underlying the behavioral change of preferences following CAT in the short and long terms. We used a general linear model (GLM) with 13 regressors: 8 regressors modeling each item type (high-value Go items, high-value NoGo items that were included in the probe task, high-value NoGo items that were not included in the probe task, high-value “sanity check” items, and the same 4 regressors for low-value items), 4 regressors modeling the mean-centered parametric modulation by subsequent probe choices (i.e., the proportion of trials each item was chosen during the subsequent probe, mean centered to ensure linear independence between these regressors and the unmodulated regressors described above), for the 4 item types, which are relevant to the Go/NoGo probe comparisons (high-value Go items, low-value Go items, high-value NoGo items that were included in the probe task, and low-value NoGo items that were included in the probe task) and one regressor for all items with a parametric modulation by the mean-centered WTP values acquired from the first BDM auction (which was added to control for initial WTP differences). These 13 regressors were convolved with the canonical double-gamma hemodynamic response function, and their temporal derivatives were added to the model. We further included at least 9 motion regressors as confounds, as described above. We estimated a model with the above described GLM regressors for each passive viewing run of each participant in a first level analysis.

In the second-level analysis (fixed effects), runs from the same session were averaged and compared with the other session. Two second-level contrasts were analyzed separately: after compared with before CAT and follow-up compared with before CAT.

All second-level analyses of all participants from after minus before or from follow-up minus before CAT were then inputted to a group level analysis (mixed effects), which included 2 contrasts of interest: one with the main effect (indicating group mean) and one with the mean centered probe effect of each participant (the demeaned proportion of choosing Go over NoGo items during the subsequent probe for the relevant pair type, i.e., high-value, low-value, or all probe pairs). The second contrast was used to test the correlation between the fMRI activations with the behavioral effect across participants. The design.fsf files (FSL format) for each participant, session, task, and analysis level can be found with the data shared on OpenNeuro.

All reported group level statistical maps were thresholded at Z > 2.3 and cluster-based Gaussian random field corrected for multiple comparisons at the whole-brain level with a (corrected) cluster significance threshold of *P =* 0.05 (Worsley 2001).

Since we found a stronger behavioral effect for high-value items, similarly to previous cue-approach samples with snack food items (Schonberg et al. 2014; Salomon et al. 2018), we focused our analyses on the contrasts for high-value items: high-value Go items, high-value Go items modulated by choice, and high-value Go minus high-value NoGo items. For completeness, we report the results of these contrasts with low-value items (low-value Go items, low-value Go items modulated by choice, and low-value Go minus low-value NoGo items), as well as a direct comparison between high-value and low-value items (i.e., high-value Go minus low-value Go items).

#### Univariate Imaging Analysis—Probe

Imaging analysis of the probe data was similar to previous imaging studies with CAT (Schonberg et al. 2014; Bakkour et al. 2017). We included 16 regressors in the model (in addition to at least 9 motion regressors as described above), based on the initial value of the probe pair (high/low) and the choice outcome (participant chose the Go/NoGo item), resulting in 4 regressors (high-value chose Go/high-value chose NoGo/low-value chose Go/low-value chose NoGo) without parametric modulation; the same 4 regressors with a parametric modulation across items by the mean-centered proportion of choices of the specific item during the entire probe phase; the same 4 regressors with a parametric modulation by the WTP difference between the 2 presented items; one regressor for all “sanity-check” trials; one regressor for all missed trials; and 2 regressors accounting for response time differences (one regressor with a modulation of the demeaned response time across trials for each value category).

Since our behavioral effect was stronger for high-value items (similar to previous cue-approach samples with snack food items), we focused our analysis on the contrasts for high-value chose Go, high-value chose Go modulated by choice and high-value chose Go minus high-value chose NoGo. Similar to analyses of the passive viewing task, we estimated a first-level GLM for each run of each participant. We then averaged the 4 runs of each probe (after/follow-up) of each participant in a second-level analysis. Finally, we ran a group level analysis as described above, with one contrast for the mean group effect and one contrast for the demeaned probe effect across participants (correlation with the behavioral effect across participants).

Some of the probe runs were excluded from the imaging analysis (i.e., not included in the second-level analysis of the specific participant) because one of the regressors was empty or because the parametric modulator of Go item choices was zeroed out, resulting in a rank-deficient design matrix. This happened, for example, when a participant chose high-value Go over high-value NoGo items on all trials of a specific run. Participants who did not have at least one full valid block (out of 2 probe blocks, each probe including one presentation of each probe pair) without any empty regressors or zeroed modulators for Go items were excluded from the probe imaging analysis. In order to minimize the number of excluded runs and participants, we did not exclude runs or participants due to a zeroed modulator of NoGo items choices but rather decided not to use the contrasts including modulation by choice of trials where NoGo items were chosen. Overall, one participant was excluded from the imaging analysis of the probe from both the after and follow-up sessions and 2 more were excluded each from one of the sessions, based on regressors causing rank-deficient matrices (in addition to the one participant that was excluded from the first session due to excessive motion, as described above). Thus, a total of 33 (out of 36) participants were included in the imaging analysis of the probe after training (out of which for 28 participants no run was excluded, for 4 participants 1 run was excluded and for 1 participant 2 runs and 1 block were excluded), and 25 (out of 27) participants were included in the imaging analysis of the follow-up probe (out of which for 21 participants no run was excluded and for 4 participants 1 run was excluded).

#### Small Volume Correction Analysis

We hypothesized that value-, attention-, and memory-related brain regions will be associated with the behavioral change following CAT: prefrontal cortex, dorsal parietal cortex, and medial-temporal lobe, respectively. Thus, in addition to the whole-brain analyses described above for the passive viewing and probe tasks, we ran similar group level analyses once for each of these prehypothesized regions (bilateral hippocampus, bilateral SPL and vmPFC), with a mask containing the voxels that were part of the region. All masks were based on the Harvard–Oxford atlas (see Supplementary Fig. 2), anatomical regions for the vmPFC mask were based on those used in previous CAT studies (Schonberg et al. 2014; Bakkour et al. 2017). The masks are shared with the data on OpenNeuro. It should be noted that the specific description of the small volume correction (SVC) analysis was unintentionally omitted from the preregistration. We further tested whether results in the SVC analysis were significant when performing Bonferroni correction for the 3 tested regions (corrected α = 0.017).

### Deviations from Preregistration

The analysis of the current work had been preceded by a preregistration describing the main methodological statistical analyses and hypothesized results of the work prior to the completion of data collection. While we aimed the preregistration to be as detailed as possible, in some parts, our description was lacking important details or was different from the eventually performed course of action. Therefore, here we describe the main differences between our preregistered analysis plan and the analyses reported in the current paper.

First, our analysis plan included analyses of diffusion MRI data, multivoxel pattern analysis, as well as generalized psychophysiological interactions (gPPI). We also scanned a control group that performed a control training task, to be used as comparison in the diffusion imaging analysis and initially planned to also be used as baseline for fMRI comparisons. We decided these analyses are beyond the scope of the current work and therefore are not reported or discussed here. Second, our preregistered analysis plan included a correct description of the first- and second-level analyses and the regressors used in our GLM model. However, it did not include a description of the planned fMRI preprocessing pipeline nor a description of the contrasts used across analyses levels. In our preregistered analysis plan, we initially planned to use FSL’s permutations tool (Randomise) for the fMRI group level analysis, and FSL’s FLAME1 as a backup (Eklund et al. 2016). Eventually, considering that FEAT’s FLAME1 might be better for our design due to within-group variance (as we specified in our preregistered analysis plan), we decided to use FLAME1. Third, functional changes in the representation of low-value items, as well as the direct comparison between the change in high-value and low-value items, were not preregistered. Nonetheless, they are reported in the results section and presented in the Supplementary Materials for completeness. Finally, our preregistration clearly indicated our prehypothesized regions of interest. Although unintentionally not directly mentioned, we preregistered these specific regions for the purpose of using them in SVC analyses, as was done in previous papers using the CAT paradigm (Schonberg et al. 2014; Bakkour et al. 2017). These regions were not used in any other unreported analysis. The intraparietal sulcus and perirhinal cortex, which were also preregistered, were not used in the SVC analysis since their anatomical definition was unclear using the Harvard–Oxford anatomical atlas.

## Results

### Behavioral Probe Results

As in previous studies with CAT, our main behavioral measure for the cue-approach effect was the proportion of Go item choices in the probe task.

#### After CAT

**Figure 2:**
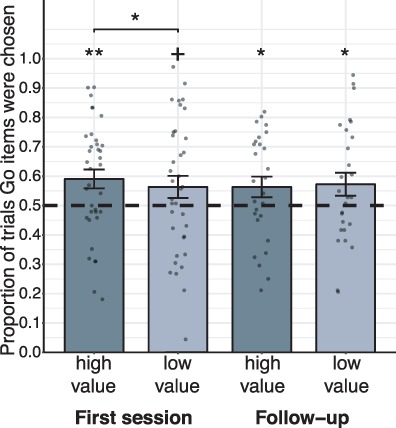
Behavioral results of Go choices during probe: mean proportion of trials in which participants chose Go over NoGo items are presented for high-value (dark gray) and low-value (light gray) probe pairs, for each session. Means of the single participants are presented with dots over each bar. The dashed line indicates chance level of 50%; error bars represent standard error of the mean. Asterisks reflect statistical significance in a one-tailed logistic regression analysis. Asterisks above each line represent proportions higher than chance (log odds = 0; odds ratio = 1). Asterisks above pairs of bars represent differential effect between the 2 value categories; +*P* < 0.1, ^*^P < 0.05, ^**^P < 0.005.

As expected from previous studies (Schonberg et al. 2014; Bakkour et al. 2016, 2017; Salomon et al. 2018) and preregistered, participants significantly preferred Go over NoGo items in high-value probe choices (mean = 0.590, SE = 0.032, *Z* = 2.823, *P =* 0.002, one-sided logistic regression) and marginally also in low-value probe choices (mean = 0.561, SE = 0.038, *Z* = 1.639, *P =* 0.051; Fig. 2). The proportion of Go items choices was significantly higher for high-value compared with low-value items (indicating a differential effect of CAT on preference for stimuli of the 2 value categories; *Z* = 2.184, *P =* 0.015, one-sided logistic regression).

#### One-Month Follow-up

One month following training (mean = 30.26 days, SD = 9.93 days, *N* = 27) participants significantly chose Go over NoGo items in both high-value (mean = 0.563, SE = 0.035, *Z* = 1.854, *P =* 0.032) and low-value (mean = 0.572, SE = 0.039, *Z* = 1.948, *P =* 0.026) probe trials. There was no differential effect between high- and low-value items in this session (*Z* = 0.622, *P =* 0.267).

### Behavioral Recognition Results

There was a prominent ceiling effect in participants’ performance in the recognition task, in both sessions. This ceiling effect did not allow us to reveal differences in memory for Go compared with NoGo items. For a full description and statistics see Supplementary analysis: behavioral recognition results.

#### Immediately After CAT

The mean hit rate across participants was 99.29% (SD = 2.08%), mean correct rejection rate of 94.16% (SD = 5.22%) and mean d′ = 3.921 (SD = 0.547). We did not find significant differences in hit rate or in RT between Go and NoGo items (see Supplementary analyses: behavioral recognition results). Results of the Go/NoGo recognition memory task showed that participants significantly remembered the associations between Go items and the cue (hit rate: mean = 79.69%, SD = 28.14%; correct rejection rate: mean = 82.65%, SD = 21.05%; d′: mean = 2.5, SD = 1.818; *P <* 0.001, 2-sided repeated measures logistic regression). Accuracy in the Go/NoGo recognition task (proportion of hits and correct rejections out of total items that are included in Go/NoGo probe choices) was significantly correlated with the proportion of Go items choices in probe for high-value items (Pearson’s *r* = 0.371, *P =* 0.026, 2-sided linear regression) but not for low-value items (Pearson’s *r* = 0.126, *P =* 0.465).

#### One-Month Follow-up

It should be noted again that the recognition task in the follow-up session was performed immediately after the passive viewing and probe tasks. Therefore, results reflect within-session memory, and not long-term memory effects from the first session. The mean hit rate was 97.81% (SD = 2.37%), mean correct rejection rate 94.26% (SD = 7.77%) and mean d′ was 3.79 (SD = 0.715). Again, we did not find significant differences in hit rate or in RT between Go and NoGo items. Results of the Go/NoGo recognition task showed that participants did not significantly remember the association between items and cues better than chance (hit rate: mean = 48.88%, SD = 31.57%; correct rejection rate: mean = 71.77%, SD = 17.95%; d′: mean = 0.56, SD = 1.61; *p =* 0.154, 2-sided logistic regression). We did not find significant correlations between accuracy in the Go/NoGo recognition task and the proportion of Go items choices in probe, neither for high-value items (Pearson’s *r* = 0.219, *P =* 0.314, 2-sided linear regression) nor for low-value items (Pearson’s *r* = 0.041, *P =* 0.853).

### Behavioral Auction Results

We ensured that our item selection procedure retained equal mean WTP between Go and NoGo items within each value category (i.e., that on average, WTP values of Go and NoGo items contrasted in probe were similar). The mean initial WTP of Go items did not significantly differ from the mean initial WTP for NoGo items, both for the high-value items (mean Go WTP: 5.758 ILS; mean NoGo WTP: 5.750 ILS; *P =* 0.960, 2-sided repeated measures logistic regression) and for the low-value items (mean Go WTP: 1.462 ILS; mean NoGo WTP: 1.478 ILS; *P =* 0.907, 2-sided repeated measures logistic regression).

Similar to previous results regarding the change in WTP following CAT (Schonberg et al. 2014), we observed a general trend of regression to the mean—that is, while WTP for high-value items significantly decreased (first session: *P =* 0.023; follow-up session: *P =* 0.002), WTP for low-value items significantly increased (*P <* 0.001 in both sessions). However, we did not find significant differences between Go and NoGo items, in both sessions (immediately after compared with before CAT and 1 month after compared with before CAT) and both value-level (high-value and low-value items). For a full description and statistics, as well as comparison between the second auction of the first session and the auction performed in the follow-up session, see Supplementary analyses: behavioral auction results.

### Imaging Results

Behavioral results with snack food items from previous studies (Schonberg et al. 2014; Bakkour et al. 2017; Salomon et al. 2018) and from the current study demonstrated a differential pattern of the change of preferences across value levels. Preference modifications were more robust for high-value compared with low-value items; therefore, we chose to focus on the functional changes in the representation of high-value items. Functional changes in the representation of low-value items, as well as the direct comparison between the change in high-value and low-value items, were not preregistered. Nonetheless, they are reported here and presented in the Supplementary Materials.

We further tested 2 kinds of relations between the behavioral effect and the neural response: “modulation across items”, meaning that the change in activity was stronger for items that were later more preferred during the subsequent probe phase (within-participant first-level parametric modulation), and “correlation across participants”, meaning that the change in activity was stronger for participants that later showed a stronger behavioral probe effect, quantified as a higher ratio of choosing Go over NoGo items (between-participants group-level correlation). Finally, for a subset of 3 prehypothesized and preregistered regions (vmPFC, hippocampus and superior parietal lobule) we performed a SVC analysis (see Materials and Methods).

All reported group level statistical maps were thresholded at *Z* > 2.3 and cluster-based Gaussian random field corrected for multiple comparisons at the whole-brain level with a (corrected) cluster significance threshold of *P =* 0.05 (Worsley 2001). Since we used 3 regions in our SVC analysis (vmPFC, hippocampus and SPL), results obtained from this analysis were examined using a Bonferroni corrected threshold of α = 0.017. Unthresholded and thresholded images of all contrasts presented here are shared on NeuroVault (Gorgolewski et al. 2015) (https://neurovault.org/collections/TTZTGQNU/).

### Passive Viewing Imaging Results

To investigate the functional changes in the response to individual items following CAT, we scanned participants with fMRI while they were passively viewing the items. Participants completed this task before, after, and 1 month following CAT (*N* = 36 before and immediately after and *N* = 27 after 1 month).

Our analysis focused on 3 main contrasts of interest: 1) regions where response to the items was enhanced after compared with before CAT; 2) regions where the correlation between the BOLD response and subsequent choices across items was changed after CAT (i.e., parametric modulation across items); and 3) regions where the response modification was weaker/stronger for participants that were more affected by CAT (i.e., correlation across participants). Contrasts 1 and 3 were tested both for Go compared with NoGo items, and for Go items separately. Contrast 2 was only tested for Go items due to potential ambiguity in interpretation of results for Go compared with NoGo items. When a specific region was found to be significant for Go items, but not for Go compared with NoGo items, we further tested whether it was also significant for NoGo items. We report all significant results for these contrasts of interest and mention when there were no significant results.

#### Immediately After Versus Before CAT (Fig. 3; for Description of All Activations See Supplementary Table 3)

**Figure 3:**
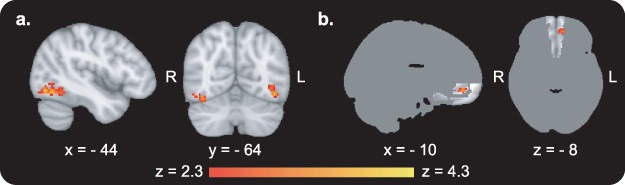
fMRI results from the passive viewing task after compared with before CAT. (*a*) Enhanced BOLD activity in bilateral occipito-temporal regions, for high-value Go compared with high-value NoGo items (whole-brain analysis). (*b*) Enhanced BOLD activity in the vmPFC in response to high-value Go items (small volume corrected results; the mask used to correct for multiple comparisons is presented on a dark gray brain silhouette). For description of all activations see Supplementary Table 3.

BOLD activity while passively viewing high-value Go compared with passively viewing high-value NoGo items was increased after compared with before CAT in the left (cluster size = 171 voxels, max *Z* value = 4.00, cluster corrected *P* = 0.014) and right (cluster size = 192 voxels, max *Z* value = 3.82, cluster corrected *P* = 0.028) occipital and temporal lobes (Fig. 3*a*), along the ventral visual processing pathway (Goodale and Milner 1992). Results of the SVC analyses revealed enhanced BOLD activity during passive viewing of high-value Go items after compared with before CAT in the vmPFC (Fig. 3*b*; cluster size = 98 voxels, max *Z* value = 3.44, cluster corrected *P* = 0.004). Activity in the vmPFC was not enhanced for NoGo items. There were no significant results for the parametric modulation across items nor for the correlation across participants immediately after compared with before CAT.

Bold activity while passively viewing low-value Go compared with passively viewing low-value NoGo items was stronger immediately after compared with before CAT in the temporo-occipital part of the left middle temporal gyrus (cluster size = 175 voxels, max *Z* value = 3.67, cluster corrected *P* = 0.021), left superior lateral occipital cortex (cluster size = 194 voxels, max *Z* value = 3.43, cluster corrected *P* = 0.011), left postcentral gyrus (cluster size = 153 voxels, max *Z* value = 3.67, cluster corrected *P* = 0.047), left posterior supramarginal/angular gyrus (cluster size = 237 voxels, max *Z* value = 4.02, cluster corrected *P* = 0.003), middle PFC (cluster size = 304 voxels, max *Z* value = 3.75, cluster corrected *P* < 0.001), and cerebellum (cluster size = 236 voxels, max *Z* value = 4.01, cluster corrected *P* = 0.003) (see Supplementary Fig. 3 and Supplementary Table 4). The significant cluster for the low-value items in the temporo-occipital visual cortex was smaller, as well as more anterior and lateral, compared with the significant cluster for the high-value items. SVC analysis in the vmPFC for low-value items immediately after compared with before CAT did not reveal any significant results.

A direct comparison between the changes for high-value Go compared with low-value Go items, as well as a comparison between the differences of high-value Go minus high-value NoGo items and the differences of low-value Go minus low-value NoGo items, revealed no significant clusters.

#### One-Month Follow-up Versus Before (Fig. 4; for Description of All Activations See Supplementary Table 5)

**Figure 4:**
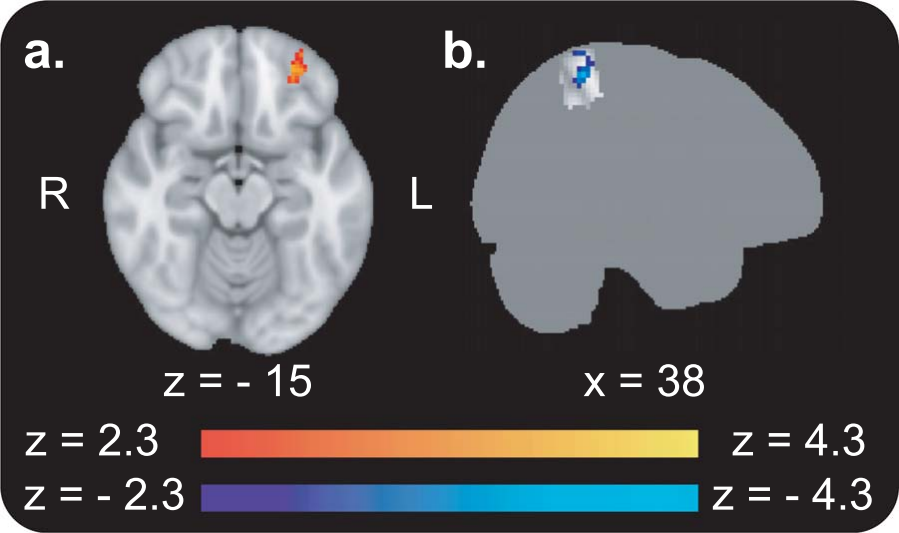
fMRI results from the passive viewing task in the 1-month follow-up compared with before CAT. (*a*) BOLD activity in response to high-value Go items in the left OFC was positively modulated by the choice effect across items (whole-brain analysis). (*b*) BOLD activity in response to high-value Go minus high-value NoGo items in the right SPL was negatively correlated with the choice effect across participants (small volume corrected). The masks used to correct for multiple comparisons in the SVC analyses are presented on a dark gray brain silhouette. For description of all activations, see Supplementary Table 5.

There were no significant whole-brain results for enhanced activity in the 1-month follow-up compared with before CAT for Go items nor for Go compared with NoGo items. BOLD activity in the left orbitofrontal cortex (OFC) in response to high-value Go items was positively modulated by the choice effect across items in the follow-up compared with before CAT (whole-brain analysis; Fig. 4*a*; cluster size = 147 voxels, max *Z* value = 3.59, cluster corrected *P* = 0.020). This modulation was not found for NoGo items. SVC analyses revealed that BOLD activity in response to high-value Go minus high-value NoGo items in the right SPL was negatively correlated with the choice effect across participants in the follow-up compared with before training (Fig. 4*b*; cluster size = 87 voxels, max *Z* value = 3.92, cluster corrected *P* = 0.009).

When observing the same contrasts for low-value items, we did not find significant changes in BOLD activity during passive viewing 1 month following compared with before CAT. A direct comparison between changes for high-value Go compared with low-value Go items also did not reveal significant differences.

Our SVC analysis revealed several activations in prehypothesized regions that were not significant when applying the more stringent Bonferroni-corrected threshold of *P* < 0.017. These results are presented in Supplementary Fig. 5 and Supplementary Table 7. A region in the vmPFC showed a trend of enhanced BOLD activity 1 month after compared with before CAT (Supplementary Fig. 5*a*; cluster size = 58 voxels, max *Z* value = 3.21, cluster corrected *P* = 0.023; not significant following Bonferroni correction), similarly to the short-term change. However, vmPFC activity was also enhanced for NoGo items (see Supplementary Fig. 4*a* and Supplementary Table 6; cluster size = 140 voxels, max *Z* value = 3.74, cluster corrected *P* < 0.001). SVC analyses further revealed that BOLD activity in response to high-value Go items in the right anterior hippocampus trended to be positively modulated by the choice effect across items in the follow-up compared with before training (Supplementary Fig. 5*b*; cluster size = 36 voxels, max *Z* value = 3.79, cluster corrected *P* = 0.045; not significant following Bonferroni correction). When testing the modulation across items in the hippocampus in response to NoGo items, we found a significant cluster in the left anterior hippocampus (see Supplementary Fig. 4*b* and Supplementary Table 6; cluster size = 62 voxels, max *Z* value = 3.67, cluster corrected *P* = 0.011).

### Probe Imaging Results

To investigate the functional response during choices, we scanned participants with fMRI while they completed the probe (binary choices) phase, as was done in previous studies (Schonberg et al. 2014; Bakkour et al. 2017). Participants completed the probe task immediately after CAT (*N* = 33). In the current study, we also scanned for the first time the probe session in the 1-month follow-up (*N* = 25).

Our analysis focused on 3 main contrasts of interest: 1) regions where response was enhanced during choices of Go compared with choices of NoGo items; 2) regions where the response was weaker/stronger for Go items that were chosen more (i.e., modulation across items). As was describes in the methods section, we were not able to test this contrast for Go minus NoGo or for NoGo items without excluding more participants, and thus only focused on this modulation for choices of Go items; 3) regions where the response was weaker/stronger for participants that were more affected by CAT (i.e., correlation across participants). This contrast was tested both for choices of Go compared with choices of NoGo items, and for choices of Go items separately. We report all significant results for these contrasts of interest and mention when there were no significant results.

#### Immediate Probe (Fig. 5*a–c*, for Description of All Activations See Supplementary Table 8)

**Figure 5:**
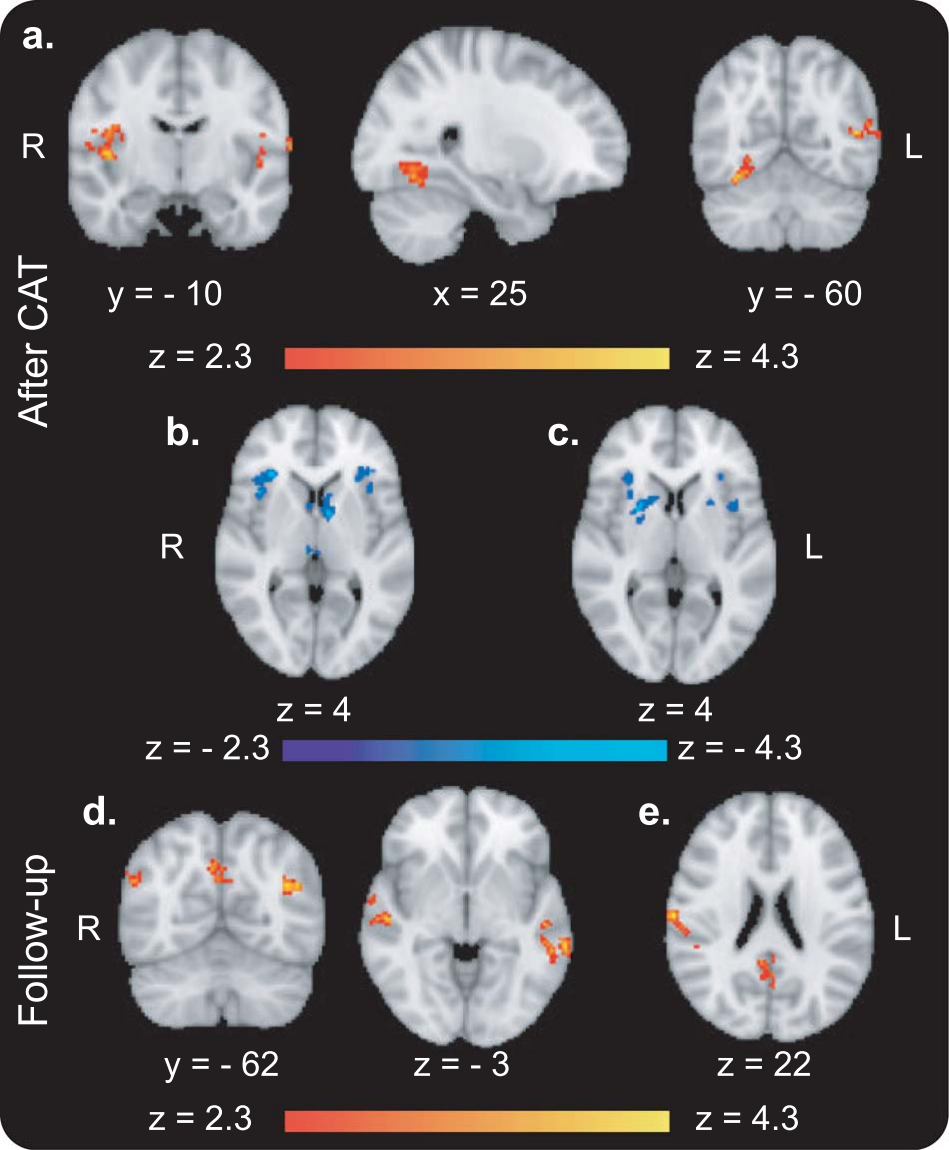
fMRI results from the probe task, immediately after and 30 days following CAT (whole-brain analysis). (*a*) Enhanced BOLD activity during choices of high-value Go compared with choices of high-value NoGo items after CAT in bilateral visual regions and bilateral central opercular cortex and Heschl’s gyrus. (*b*) BOLD response after CAT was negatively correlated with the choice effect across participants and (*c*) negatively modulated by the choice effect across items, during choices of high-value Go over high-value NoGo items in the striatum as well as other regions. (*d*) Bold activity during choices of high-value Go items in the 1-month follow-up was positively modulated by the choice effect across items in the precuneus, bilateral superior occipital cortex, and bilateral middle and superior temporal gyrus and (*e*) positively correlated with the choice effect across participants in the precuneus/posterior cingulate cortex (PCC) and right post-central gyrus. For description of all activations see Supplementary Table 8 and Supplementary Table 10.

BOLD activity was stronger during choices of high-value Go over high-value NoGo items compared with choices of high-value NoGo over high-value Go items in bilateral visual regions (right: cluster size = 131 voxels, max *Z* value = 3.86, *P* = 0.048; left: cluster size = 188 voxels, max *Z* value = 4.04, cluster corrected *P* < 0.001) and bilateral central opercular cortex and Heschl’s gyrus (right: cluster size = 152 voxels, max *Z* value = 3.91,P*p* = 0.020; left: cluster size = 136 voxels, max Z value = 3.74, cluster corrected *P* = 0.039) (Fig. 5*a*). In addition, BOLD activity in the striatum while choosing high-value Go compared with choosing high-value NoGo items after CAT was negatively correlated with the choice effect across participants (the ratio of choosing high-value Go items during probe; cluster size = 148 voxels, max *Z* value = 4.00, cluster corrected *P* = 0.024; Fig. 5*b*) and negatively modulated by the choice affect across items (cluster size = 245 voxels, max *Z* value = 3.88, cluster corrected *P* < 0.001; Fig. 5*c*).

There were no regions with significant stronger BOLD activity during choices of low-value Go items compared with choices of low-value NoGo items after CAT. In the superior division of the left lateral occipital cortex, the difference between activity during choices of low-value Go items and activity during choices of low-value NoGo items was negatively correlated with the choice effect across participants (cluster size = 155 voxels, max *Z* value = 3.43, cluster corrected *P* = 0.009; see Supplementary Fig. 6*a*). In addition, BOLD activity in the left (cluster size = 260 voxels, max *Z* value = 3.82, cluster corrected *P* < 0.001) and right (cluster size = 352 voxels, max *Z* value = 4.24, cluster corrected *P* < 0.001) Heschl’s gyrus/central opercular cortex and in the left precuneous (cluster size = 158 voxels, max *Z* value = 3.62, cluster corrected *P* = 0.016) was stronger immediately after CAT during choices of high-value Go items compared with choices of low-value Go items (see Supplementary Fig. 6b and Supplementary Table 9).

Our SVC analysis revealed several activations in prehypothesized regions that were not significant when applying the more stringent Bonferroni-corrected threshold of *P* < 0.017. These results are presented in Supplementary Fig. 5. BOLD activity in the right SPL while choosing high-value Go items after CAT demonstrated a trend of negative correlation with the choice effect across participants (Supplementary Fig. 5*c*; cluster size = 53 voxels, max *Z* value = 3.44, cluster corrected *P* = 0.035, not significant following Bonferroni correction) and a trend of negative modulation by the choice effect across items (Supplementary Fig. 5*d*; cluster size = 47 voxels, max *Z* value = 3.80, cluster corrected *P* = 0.048, not significant following Bonferroni correction).

#### One-Month follow-up Probe (Fig. 5*d,e*, for Description of All Activations See Supplementary Table 10)

In the follow-up probe, we did not find regions where BOLD activity during choices of high-value Go items was stronger than during choices of NoGo items. BOLD activity in the precuneus (cluster size = 151 voxels, max *Z* value = 3.67, cluster corrected *P* = 0.005), bilateral superior occipital cortex (right: cluster size = 208 voxels, max *Z* value = 3.87, cluster corrected *P <* 0.001; left: cluster size = 201 voxels, max *Z* value = 4.18, cluster corrected *P* < 0.001) and bilateral middle and superior temporal gyrus (right: cluster size = 175 voxels, max *Z* value = 3.89, cluster corrected *P* = 0.002; left: cluster size = 342 voxels, max *Z* value = 4.3, cluster corrected *P* < 0.001) while choosing high-value Go items in the follow-up probe was positively modulated by the choice effect across items (Fig. 5*d*). BOLD activity in the precuneus/posterior cingulate cortex (PCC; cluster size = 474 voxels, max *Z* value = 3.80, cluster corrected *P* < 0.001) and right post-central gyrus (cluster size = 181 voxels, max *Z* value = 4.40, cluster corrected *P* = 0.004) while choosing high-value Go items in the follow-up probe was positively correlated with the choice effect across participants (Fig. 5*e*). There were no significant results for these contrasts for low-value items. However, BOLD activity in the left (cluster size = 265 voxels, max *Z* value = 3.98, cluster corrected *P* < 0.001) and right (cluster size = 588 voxels, max *Z* value = 4.25, cluster corrected *P* < 0.001) occipital poles was stronger 1 month after CAT during choices of high-value Go items compared with choices of low-value Go items (see Supplementary Fig. 6*c* and Supplementary Table 9).

## Discussion

In the current work, we set out to examine the neural mechanisms underlying nonreinforced behavioral change following CAT, in the short and in the long term. We introduced a novel passive viewing task to study the functional plasticity of response to single items before, after and 1 month following CAT. We predicted and preregistered that the underlying neural mechanisms will involve specific memory, attention, and value-related brain regions.

Behaviorally, our main hypothesis was that we will replicate the CAT effect, that is, participants will significantly choose high-value cued (high-value Go) over high-value noncued (high-value NoGo) items, both immediately and 1 month following CAT. In addition, we hypothesized better memory for Go compared with NoGo items, a correlation between this memory difference and the CAT effect and potentially a weaker regression to the mean of the WTP for Go compared with NoGo items.

The behavioral results obtained in the current study (see Fig. 2) replicated previous results and in accordance with our preregistered predictions demonstrated enhanced preferences toward high-value Go compared with high-value NoGo items following CAT (Schonberg et al. 2014; Bakkour et al. 2016, 2017, 2018; Veling et al. 2017; Zoltak et al. 2017; Salomon et al. 2018).

The fMRI results of this study suggest the involvement of several neural components in preference modification induced by CAT.

### Perceptual Enhancement in the Short Term

Examining the neural response for high-value Go compared with high-value NoGo items following CAT revealed enhanced processing in ventral visual regions (Fig. 3*a*). We refer to these changes as “bottom-up” processes to emphasize that they occurred in perceptual regions, contrary to the common view, which localizes value-based decision-making in high-level prefrontal regions. We also found an indication for enhancement of visual processing in response to low-value Go items following CAT (Supplementary Fig. 3). This enhancement was in a smaller region, which might be related to the weaker behavioral effect for low-value items. There were no significant results in a direct comparison between the change of response to high-value Go versus low-value Go items. However, it should be noted that our study was not well powered for this comparison, since we focused our analyses on the high-value items, for which there was a stronger behavioral change, both in this study and previous ones with snack food items (Schonberg et al. 2014; Salomon et al. 2018). Eye gaze recorded during the passive viewing task from a subset of participants did not reveal longer gaze duration on paired items (see Supplementary exploratory analysis: eye tracking) immediately after training and thus is probably not the reason for the enhanced fMRI signal for high-value Go over NoGo.

We show here for the first time that activity in high-level visual processing occipito-temporal cortex was involved when preferences were modified using CAT, without external reinforcements. Activity in low- and high-level visual regions was previously shown to be related to past rewards (Serences 2008) and to value of visual attributes later integrated to overall stimulus value in the vmPFC (Lim et al. 2013), but not to preferences modification in the absence of external reinforcements as in the current study. We suggest that the functional changes in visual regions reflect modifications in the perceptual representation of the paired items (Ishai et al. 2000; Grill-Spector 2003). In the short-term, the enhanced bottom-up processing and representation change of individual Go items putatively lead to enhanced value-related processing and enhanced preferences toward these items during choices.

### Decreased Top-Down Attention

Participants with overall greater long-term behavioral change demonstrated reduced change of response to high-value Go items in the follow-up compared with before CAT in the right SPL (i.e., activity was negatively correlated with the choice effect across participants in the long term; see Fig. 4*b*). This finding suggests reduced involvement of top-down parietal mechanisms during passive viewing of Go compared with NoGo items (Culham and Kanwisher 2001). This finding is in the opposite direction of our preregistered prediction of enhanced involvement of attention-related mechanisms. Decreased top-down attentional mechanisms may underlie the impulsive-like nature of the preference bias toward Go items (Veling et al. 2017).

### Putative Long-Term Maintenance via Memory Processes, Based on Marginal Results

We preregistered our hypothesis that hippocampal memory processes will be involved in CAT. SVC analysis of the passive viewing task 1 month after compared with before CAT showed a trend of enhanced hippocampal activity modulated by the proportion of trials. Go items were chosen in the subsequent probe task. This trend however did not exceed statistical significance after Bonferroni correction for number of regions (see Supplementary Fig. 5*b*). The same contrast was significant for high-value NoGo items (see Supplementary Fig. 4*b*). These findings suggest that items, mainly NoGo items, that are more recognized or more vividly attended to during passive viewing are later chosen more often.

These results lead to 2 possible interpretations: in one, the hippocampal modulated activity suggests that CAT, putatively through immediate perceptual processing enhancement, affected the encoding and accessibility of items and their related associations in memory after 30 days, which in turn affected choices. Alternatively, it is possible that the relation between long-term memory enhancement and choices is the result of a mere-choice effect (Ariely and Norton 2008; Sharot et al. 2010, 2012), as the binary probe task was performed in the first session immediately after CAT. The short-term effect can only be attributed to CAT, while the long-term effect may involve a choice effect. Importantly, even if the long-term effect is partially the result of a choice effect (which the current study cannot test), it still holds great promise as a behavioral intervention for long-lasting behavioral change. Since the parametric modulation in the hippocampus for high-value Go items was not significant with the Bonferroni-corrected threshold, while the modulation for high-value NoGo items was, the interaction between memory processes and the behavioral effect may be stronger for NoGo items than for Go items, putatively due to other processes being involved in the enhanced preferences toward high-value Go items.

Although participants completed a behavioral recognition task at the end of the experiment, this experiment was not designed to test behavioral memory modifications. Therefore, the recognition task was performed immediately after the rest of the tasks. This design resulted in a ceiling effect of over 99% hit rate across participants, masking any memory differences between Go and NoGo items. Differences in RT were also not optimal for testing, as the task was self-paced. Moreover, in the follow-up session, the recognition task was again performed following the probe task; therefore, long-term memory for the items (old/new) was not actually tested.

The Go/NoGo recognition task tested participants’ memory for the cue-item associations. Unlike the old/new recognition task, this task did measure long-term memory, since training was only performed in the first session. However, the Go/NoGo recognition task only tested memory of the cue-item associations, while the old/new recognition task measured general recognition memory of the items. Results of this task showed that participants significantly remembered the association of Go items with the cue immediately after CAT, but not in the 1-month follow-up. The correlation between recognizing Go items and choosing Go over NoGo items was only significant for high-value items in the first session. These findings raise the possibility that the stronger response to Go items in high-level perceptual regions was related to participants recognizing the high-value Go items only immediately, but not 1 month after CAT. However, since the recognition task was performed after choices, recognition memory could have been affected by previous choices.

Overall, our behavioral recognition data do not allow us to behaviorally test for memory involvement in the short- or long-term effect of CAT. Future behavioral studies, designed specifically to test for memory modifications, are needed to explore the involvement of memory processes in the behavioral change following CAT, both in the short and long term, as well as to test the involvement of a choice effect in the long term. This can be done for example by omitting the immediate probe task and testing choices only in the follow-up session.

### Enhanced Value Response

Value change is reflected in enhanced neural response of the vmPFC to high-value Go items, immediately after CAT (see Fig. 3*b*) and marginally in the 1-month follow-up (see Supplementary Fig. 5*a*; note that this result was not significant when using a Bonferroni-corrected threshold). In the 1-month follow-up, value change was further reflected in the OFC, where activity was stronger while passively viewing high-value Go items that were later chosen more during the subsequent probe phase (see Fig. 4*a*). These findings may indicate, as predicted and preregistered, a long-lasting value change signature of individual items not during choices (Lebreton et al. 2009; Levy et al. 2011). Overall, these results reveal for the first time an item-level value change during passive viewing (Serences 2008; Levy et al. 2011), in line with previous findings of enhanced activity in the vmPFC during binary choices of more preferred high-value Go items (Schonberg et al. 2014). It should be noted that we also found enhancement of vmPFC activity in the follow-up compared with before CAT during passive viewing of high-value NoGo items. This nonselective enhancement may be the result of a mere exposure effect (Zajonc 1968), where the value of items is enhanced following repeated exposure to them (although it was found for NoGo items only in the follow-up and not immediately after CAT).

We suggest that the joint effect of the 3 above-described components provides a shift toward enhanced bottom-up over top-down processes, resulting in non reinforced behavioral change that can be maintained for a long period of time. Based on the findings of the current study, we propose that the low-level association of visual, auditory, and motor systems during training modifies valuation of items via a network including enhanced bottom-up perceptual processes in the immediate short term, which is translated to long-term maintenance by decrease of top-down attentional control and putatively memory enhancement. This leads to long-lasting behavioral change. In Supplementary Fig. 7 we provide a putative outline of the suggested dynamics of this preference change process. Although the proposed model dynamics are based on reverse inference from the current work data, its principal components (i.e., top-down attention-, memory-, and value-related mechanisms) were predicted and preregistered prior to analyses. The methods and results of our current study do not lend themselves to directly testing this suggested mechanism using current available methods. Future studies should test the validity and reproducibility of these results using independent data.

### Functional Activity During Binary Choices also Reflect Enhanced Bottom-Up and Decreased Top-Down Mechanisms of Preferences Modification

Functional MRI responses during binary choices also resonate the proposed dynamics for the nonreinforced behavioral change (Supplementary Fig. 7), demonstrating enhanced perceptual processing in the short-term and marginal evidence of involvement of memory processes as well as decreased top-down attention mechanisms in the long-term.

When participants chose high-value Go over high-value NoGo items, activity in perceptual regions—both visual and auditory—was enhanced (see Fig. 5*a*). These findings suggest that, in the short-term, retrieval of the low-level visual and auditory associations constructed during training were associated with choices of Go items. Thus, functional response during choices further supports the involvement of enhanced bottom-up processing in the immediate nonreinforced modification of preferences.

In the follow-up session, choices of high-value Go over NoGo items were associated with enhanced BOLD activity in the precuneus and PCC (see Fig. 5*d,e*), which have been related to episodic memory retrieval and are also considered to be part of the default mode network (Shallice et al. 1994; Fransson and Marrelec 2008; Sestieri et al. 2011). This provides additional support for the putative role of memory processes in the long-term retention of the behavioral change.

Although not significant after the Bonferroni-corrected threshold, uncorrected results from the probe SVC analysis provide limited support for the involvement of reduced top-down attentional mechanisms revealed from the passive viewing task. During choices of high-value Go items, activity in the SPL showed a trend of negative modulation by the choice effect across items as well as negatively correlated with the choice effect across participants (though in a more posterior and inferior region, see Supplementary Fig. 5*c,d*).

We were not able to replicate previous results showing enhanced activity in the vmPFC during choices of Go items that were chosen more overall (Schonberg et al. 2014; Bakkour et al. 2017). These previous results were found for high-value Go items when the group’s behavioral effect of choosing high-value Go items was significant but weak relative to other samples (study 3 in Schonberg et al. 2014). Similar results were found for choices of low-value Go compared with choices of low-value NoGo items, and not for choices of high-value Go items, when the behavioral effect was strong for high-value items and weak for low-value items (Bakkour et al. 2017). Therefore, a possible account for the lack of replication of these findings in the current study is that this contrast of modulation across items depends on the variance of the choice effect across items, which seems to be smaller in the current study compared with previous samples that found this effect.

Overall, neural activity during binary choices support our suggested proposal of nonreinforced behavioral change (Supplementary Fig. 7), demonstrating similar patterns to these shown in the passive viewing task: enhanced perceptual processing in the short-term and putatively long-term manifestation of the behavioral change through reduced top-down involvement (here both in the short and long term) and memory-related mechanisms.

### Additional Behavioral Findings

As in Salomon et al. (2018), we found a significant correlation between choices across sessions: items that were chosen in the first session were more likely to be chosen again in the follow-up session. These results offer 2 possible interpretations: first, that CAT enhanced preferences of specific items, and these effects last for months, or second, that CAT affected immediate choices and follow-up choices were consistent with immediate choices via a “mere choice” effect (Ariely and Norton 2008; Sharot et al. 2010, 2012) or an effect on memory processes. The design of the current study does not enable us to differentiate between these 2 possibilities. Future studies could better resolve these 2 alternative hypotheses by testing the long-term maintenance of the CAT effect without performing a probe choice immediately after training. Importantly, as the immediate effect is the direct result of CAT, both possibilities are interesting and reflect the great potential of CAT as a behavioral intervention for long-lasting behavioral change.

Similar to previous experiments (Schonberg et al. 2014), participants completed a BDM auction task at the beginning of the experiment as well as at the end of each session. Previous CAT experiments identified regression to the mean for both Go and NoGo items (Schonberg et al. 2014). One previous experiment found regression to the mean to be weaker for Go compared with NoGo items, but a different experiment in the same study did not replicate this effect (Schonberg et al. 2014). In the current study, we found regression to the mean with no differences between Go and NoGo items (i.e., no effect of CAT on WTP). Although the second auction was performed after participants knew they will get one item at the end of the experiment based on a single random choice from the probe task, we suggest that the auction still validly measured WTP for 2 reasons: 1) at the time of the second auction participants did not know which item they will receive based on the probe task (which could potentially be a low-valued one); 2) participants were strongly influenced by the long food-abstinence manipulation, including 4 h of fasting prior to the experiment and the additional time of completing the experiment.

The results of the WTP comparison between auctions suggest 2 main hypotheses: first, that CAT affects choices, not via a change in WTP but rather via other mechanisms that are involved in forced choices but not monetary valuation. Or second, that a single WTP test following CAT was not sensitive enough to elicit a behavioral effect, while a forced binary choice paradigm between similarly valued stimuli could have detected value modification effects more reliably.

Exploratory analysis testing RTs in the probe task (see Supplementary exploratory analyses: probe choice response times) revealed that choices of Go items were significantly faster than choices of NoGo items in both sessions. A previous study also found that healthy participants make probe Go choices faster than probe NoGo choices (Aridan et al. 2019). This finding may indicate either that the value of Go items was enhanced and thus choices of these now more valued items are faster (Krajbich et al. 2010; Milosavljevic et al. 2010) or that Go item choices are more impulsive and are therefore faster (Veling et al. 2017).

### Limitations and Additional Considerations

Our sample size was based on a power analysis performed with data of a previous finding of enhanced vmPFC activity during choices of high-value Go versus high-value NoGo items modulated by the number of times each item was chosen (Schonberg et al. 2014). Therefore, although our study focused on the novel passive viewing task, the power analysis was an estimation based on the probe analysis rather than the novel passive viewing analysis. Current power analysis methods are based on previous results or a known effect size in a specific region (e.g., http://www.fmripower.org/ and http://neuropowertools.org/neuropower/neuropowerstart/). Furthermore, these methods do not account for the effect of whole-brain (or small-volume) cluster correction analysis. Therefore, it should be noted that, similar to many other fMRI studies, we cannot indicate with certainty whether we are sufficiently powered to detect the different effects that were tested. Nonetheless, we reported results for all performed analyses and highlighted the significant results while not claiming null effects.

In our imaging analysis we tested several contrasts of interest. Generally, we tested 1) in which regions the response to the items was changed following CAT, 2) in which regions the change in response was modulated by subsequent choices across items, and 3) in which regions the change in response was correlated with the behavioral effect across participants. The first contrast examined overall change in response to all Go items. Results found in this contrast for Go versus NoGo items (e.g., the enhancement in visual regions) are considered stronger than results found only for Go items or both for Go and NoGo items (e.g., the enhanced response in vmPFC). The second contrast of interest was directly related to behavior and directly tested the regions where the change in response was related to choices of specific items within participants. The third contrast of interest tested for regions which were related to behavioral differences across participants, as CAT is a group-level effect with some participants showing stronger effect than others.

While a region showing both an overall change for Go and not for NoGo items after CAT and a correlation with the behavioral effect would have been considered most related to the CAT effect, our findings immediately after CAT were found in contrasts testing for main effects across all high-value paired items, whereas results from the 1-month follow-up were obtained mainly in parametric modulation of choices across items (within participants). This might be explained by the nature of CAT as a group effect. Not all participants significantly choose Go over NoGo items following training and there is considerable variance across participants. In addition, training is performed at the individual item level, and thus preferences might be affected only for some but not all paired items.

The CAT effect in the 1-month follow-up was relatively weak in the current study, although the behavioral effect was found to last for months in previous studies (Schonberg et al. 2014; Salomon et al. 2018). Since the long-term behavioral effect was weaker than the effect in the short term, the variance across items was larger and putatively enabled us to find differences in the response to different items in the follow-up session. The weaker long-term effect may have been the result of the new passive viewing task. In this task, which was completed before, after and 1 month after training, items were presented on the screen without cues or motor responses, possibly resulting in partial extinction of the cue-item pairing established during training.

Our results suggest that, in the short term, perceptual processing is enhanced beyond all paired items, while in the long term, the effect persists only for some of the items; thus the overall behavioral effect is weaker and the neural changes are more prominent for the items that elicited weaker response in top-down attention-related regions.

## Conclusions

Research of value-based decision-making and behavioral change interventions focused on top-down mechanisms such as self-control or external reinforcements as the main means to change preferences (Rangel et al. 2008; Wood and Neal 2016). The CAT paradigm has been shown to change preferences using the mere association of images of items with a cued speeded button response without external reinforcements. The paradigm is highly replicable with dozens of studies demonstrating the ability to change behavior for months with various stimuli and cues (Schonberg et al. 2014; Bakkour et al. 2016, 2017, 2018; Veling et al. 2017; Zoltak et al. 2017; Salomon et al. 2018). Current interventions that rely on reinforcement and self-control fail to change behavior for the long term. Our findings emphasize the importance and great potential of targeting bottom-up rather than top-down mechanisms to induce long-lasting behavioral change. Our uncorrected results hint at an involvement of memory processes in value-based decision-making and its relevance to the durability of the behavioral change, which should be directly tested in future studies. We present a suggested model for the dynamics underlying this change. Our current findings can lead to new theories relating perceptual processing, attention, and putatively memory to preferences and decision-making. They hold promise for new long-term behavioral change interventions targeting this novel pathway for value change based on bottom-up mechanisms, which can lead to long lasting change and thus improve the quality of life for people around the world.

## Funding

European Research Council (under the European Union’s Horizon 2020 research and innovation programme; 715016 to T.SC.); Israel Science Foundation (to T.SC.) T.Sa was supported by the Nehemia Levtzion fellowship.

## Supplementary Material

BotvinikNezer_CerebralCortex_final_submission_supp_bhz132

## Notes

We thank Dr Jeanette Mumford for her invaluable statistics advices.

## References

[ref1] Aridan N, Pelletier G, Fellows LK, Schonberg T 2019 Is ventromedial prefrontal cortex critical for behavior change without external reinforcement? Neuropsychologia. 124:208–215. 30550808 10.1016/j.neuropsychologia.2018.12.008 PMC6372830

[ref2] Ariely D, Norton MI 2008 How actions create—not just reveal—preferences. Trends Cogn Sci. 12:13–16. 18063405 10.1016/j.tics.2007.10.008

[ref3] Avants BB, Epstein CL, Grossman M, Gee JC 2008 Symmetric diffeomorphic image registration with cross-correlation: evaluating automated labeling of elderly and neurodegenerative brain. Med Image Anal. 12:26–41. 17659998 10.1016/j.media.2007.06.004 PMC2276735

[ref4] Bakkour A, Botvinik-Nezer R, Cohen N, Hover AM, Poldrack RA, Schonberg T 2018 Spacing of cue-approach training leads to better maintenance of behavioral change. PLoS One. 13:1–39. 10.1371/journal.pone.0201580 PMC6066248 30059542

[ref5] Bakkour A, Leuker C, Hover AM, Giles N, Poldrack RA, Schonberg T 2016 Mechanisms of choice behavior shift using cue-approach training. Front Psychol. 7:1–12. 27047435 10.3389/fpsyg.2016.00421 PMC4804288

[ref6] Bakkour A, Lewis-Peacock JA, Poldrack RA, Schonberg T 2017 Neural mechanisms of cue-approach training. Neuroimage. 151:92–104. 27677231 10.1016/j.neuroimage.2016.09.059 PMC5365383

[ref7] Becker GM, DeGroot MH, Marschak J 1964 Measuring utility by a single-response sequential method. Behav Sci. 9:226–232. 5888778 10.1002/bs.3830090304

[ref8] Brown TI, Staresina BP, Wagner AD 2015 Noninvasive functional and anatomical imaging of the human medial temporal lobe. Cold Spring Harb Perspect Biol. 7:a021840. 25780085 10.1101/cshperspect.a021840 PMC4382742

[ref9] Cabeza R, Ciaramelli E, Olson IR, Moscovitch M 2008 The parietal cortex and episodic memory: an attentional account. Nat Rev Neurosci. 9:613–625. 18641668 10.1038/nrn2459 PMC2692883

[ref10] Chib VS, Rangel A, Shimojo S, O’Doherty JP 2009 Evidence for a common representation of decision values for dissimilar goods in human ventromedial prefrontal cortex. J Neurosci. 29:12315–12320. 19793990 10.1523/JNEUROSCI.2575-09.2009 PMC6666137

[ref11] Christiansen T, Bruun JM, Madsen EL, Richelsen B 2007 Weight loss maintenance in severely obese adults after an intensive lifestyle intervention: 2- to 4-year follow-up. Obesity (Silver Spring). 15:413–420. 17299115 10.1038/oby.2007.530

[ref12] Corbetta M, Shulman GL 2002 Control of goal-directed and stimulus-driven attention in the brain. Nat Rev Neurosci. 3:215–229. 10.1038/nrn755 11994752

[ref13] Culham JC, Kanwisher NG 2001 Neuroimaging of cognitive functions in human parietal cortex. Curr Opin Neurobiol. 11:157–163. 11301234 10.1016/s0959-4388(00)00191-4

[ref14] Dale AM, Fischl B, Sereno MI 1999 Cortical surface-based analysis: I. segmentation and surface reconstruction. Neuroimage. 9:179–194. 9931268 10.1006/nimg.1998.0395

[ref15] Deichmann R, Gottfried JA, Hutton C, Turner R 2003 Optimized EPI for fMRI studies of the orbitofrontal cortex. Neuroimage. 19:430–441. 12814592 10.1016/s1053-8119(03)00073-9

[ref16] Eklund A, Nichols TE, Knutsson H 2016 Cluster failure: why fMRI inferences for spatial extent have inflated false-positive rates. Proc Natl Acad Sci. 113:7900–7905. 27357684 10.1073/pnas.1602413113 PMC4948312

[ref17] Esteban O, Markiewicz CJ, Blair RW, Moodie CA, Isik AI, Erramuzpe A, Kent JD, Goncalves M, DuPre E, Snyder M et al. 2019 fMRIPrep: a robust preprocessing pipeline for functional MRI. Nat Methods. 16:111–116. 30532080 10.1038/s41592-018-0235-4 PMC6319393

[ref18] Fonov VS, Evans AC, McKinstry RC, Almli CRCD 2009 Unbiased nonlinear average age-appropriate brain templates from birth to adulthood. Neuroimage. 54(1): 313–327. 10.1016/j.neuroimage.2010.07.033 PMC2962759 20656036

[ref19] Fransson P, Marrelec G 2008 The precuneus/posterior cingulate cortex plays a pivotal role in the default mode network: evidence from a partial correlation network analysis. Neuroimage. 42:1178–1184. 18598773 10.1016/j.neuroimage.2008.05.059

[ref20] Goodale MA, Milner AD 1992 Separate visual pathways for perception and action. Trends Neurosci. 15:20–25. 1374953 10.1016/0166-2236(92)90344-8

[ref21] Gorgolewski KJ, Auer T, Calhoun VD, Craddock RC, Das S, Duff EP, Flandin G, Ghosh SS, Glatard T, Halchenko YO et al. 2016 The brain imaging data structure, a format for organizing and describing outputs of neuroimaging experiments. Sci Data. 3:1–9. 10.1038/sdata.2016.44 PMC4978148 27326542

[ref22] Gorgolewski KJ, Varoquaux G, Rivera G, Schwarz Y, Ghosh SS, Maumet C, Sochat VV, Nichols TE, Poldrack RA, Poline J-B et al. 2015 NeuroVault.Org: a web-based repository for collecting and sharing unthresholded statistical maps of the human brain. Front Neuroinform. 9:1–9. 25914639 10.3389/fninf.2015.00008 PMC4392315

[ref23] Grill-Spector K 2003 The neural basis of object perception. Curr Opin Neurobiol. 13:159–166. 12744968 10.1016/s0959-4388(03)00040-0

[ref24] Ishai A, Ungerleider LG, Martin A, Haxby JV 2000 The representation of objects in the human occipital and temporal cortex. J Cogn Neurosci. 12(Suppl 2):35–51. 11506646 10.1162/089892900564055

[ref25] Jeffery RW, Drewnowski a ELH, Stunkard a J, Wilson GT, Wing RR, Hill DR 2000 Long-term maintenance of weight loss: current status. Health Psychol. 19:5–16. 10709944 10.1037/0278-6133.19.suppl1.5

[ref26] Kable JW, Glimcher PW 2009 The neurobiology of decision: consensus and controversy. Neuron. 63:733–745. 19778504 10.1016/j.neuron.2009.09.003 PMC2765926

[ref27] Krajbich I, Armel C, Rangel A 2010 Visual fixations and the computation and comparison of value in simple choice. Nat Neurosci. 13:1292–1298. 20835253 10.1038/nn.2635

[ref28] Lebreton M, Jorge S, Michel V, Thirion B, Pessiglione M 2009 An automatic valuation system in the human brain: evidence from functional neuroimaging. Neuron. 64:431–439. 19914190 10.1016/j.neuron.2009.09.040

[ref29] Levy I, Lazzaro SC, Rutledge RB, Glimcher PW 2011 Choice from non-choice: predicting consumer preferences from blood oxygenation level-dependent signals obtained during passive viewing. J Neurosci. 31:118–125. 21209196 10.1523/JNEUROSCI.3214-10.2011 PMC3078717

[ref30] Li X, Morgan PS, Ashburner J, Smith J, Rorden C 2016 The first step for neuroimaging data analysis: DICOM to NIfTI conversion. J Neurosci Methods. 264:47–56. 26945974 10.1016/j.jneumeth.2016.03.001

[ref31] Lim S-L, O’Doherty JP, Rangel A 2013 Stimulus value signals in ventromedial PFC reflect the integration of attribute value signals computed in fusiform gyrus and posterior superior temporal gyrus. J Neurosci. 33:8729–8741. 23678116 10.1523/JNEUROSCI.4809-12.2013 PMC3865515

[ref32] Marteau TM, Hollands GJ, Fletcher PC 2012 Changing human behavior to prevent disease: the importance of targeting automatic processes. Science. 337:1492–1495. 22997327 10.1126/science.1226918

[ref33] Milosavljevic M, Malmaud J, Huth A, Koch C, Rangel A 2010 The drift diffusion model can account for the accuracy and reaction time of value-based choices under high and low time pressure. Judgm Decis Mak. 5:437–449.

[ref34] Moeller S, Yacoub E, Olman CA, Auerbach E, Strupp J, Harel N, Uğurbil K 2010 Multiband multislice GE-EPI at 7 tesla, with 16-fold acceleration using partial parallel imaging with application to high spatial and temporal whole-brain FMRI. Magn Reson Med. 63:1144–1153. 20432285 10.1002/mrm.22361 PMC2906244

[ref35] Padoa-Schioppa C 2011 Neurobiology of economic choice: a good-based model. Annu Rev Neurosci. 34:333–359. 21456961 10.1146/annurev-neuro-061010-113648 PMC3273993

[ref36] Prochaska JJ, Delucchi K, Hall SM 2004 A meta-analysis of smoking cessation interventions with individuals in substance abuse treatment or recovery. J Consult Clin Psychol. 72:1144–1156. 15612860 10.1037/0022-006X.72.6.1144

[ref37] Rangel A, Camerer C, Montague PR 2008 A framework for studying the neurobiology of value-based decision making. Nat Rev Neurosci. 9:545–556. 18545266 10.1038/nrn2357 PMC4332708

[ref38] Salomon T, Botvinik-Nezer R, Gutentag T, Gera R, Iwanir R, Tamir M, Schonberg T 2018 The cue-approach task as a general mechanism for long-term non-reinforced behavioral change. Sci Rep. 8:3614. 29483525 10.1038/s41598-018-21774-3 PMC5827734

[ref39] Schonberg T, Bakkour A, Hover AM, J a M, Nagar L, Perez J, R a P 2014 Changing value through cued approach: an automatic mechanism of behavior change. Nat Neurosci. 17:625–630. 24609465 10.1038/nn.3673 PMC4041518

[ref40] Serences JT 2008 Value-based modulations in human visual cortex. Neuron. 60:1169–1181. 19109919 10.1016/j.neuron.2008.10.051 PMC3384552

[ref41] Sestieri C, Corbetta M, Romani GL, Shulman GL 2011 Episodic memory retrieval, parietal cortex, and the default mode network: functional and topographic analyses. J Neurosci. 31:4407–4420. 21430142 10.1523/JNEUROSCI.3335-10.2011 PMC3098040

[ref42] Shadlen MN, Shohamy D 2016 Decision making and sequential sampling from memory. Neuron. 90:927–939. 27253447 10.1016/j.neuron.2016.04.036 PMC4891701

[ref43] Shallice T, Fletcher P, Frith CD, Grasby P, Frackowiak RS, Dolan RJ 1994 Brain regions associated with acquisition and retrieval of verbal episodic memory. Nature. 368:633–635. 8145849 10.1038/368633a0

[ref44] Sharot T, Fleming SM, Yu X, Koster R, Dolan RJ 2012 Is choice-induced preference change long lasting? Psychol Sci. 23:1123–1129. 22933456 10.1177/0956797612438733 PMC3802118

[ref45] Sharot T, Velasquez CM, Dolan RJ 2010 Do decisions shape preference? Psychol Sci. 21:1231–1235. 20679522 10.1177/0956797610379235 PMC3196841

[ref46] Shohamy D, Daw ND 2015 Integrating memories to guide decisions. Curr Opin Behav Sci. 5:85–90.

[ref47] Smith SM, Jenkinson M, Woolrich MW, Beckmann CF, Behrens TEJ, Johansen-Berg H, Bannister PR, De Luca M, Drobnjak I, Flitney DE et al. 2004 Advances in functional and structural MR image analysis and implementation as FSL. Neuroimage. 23:208–219. 10.1016/j.neuroimage.2004.07.051 15501092

[ref48] Veling H, Chen Z, Tombrock MC, Verpaalen IAM, Schmitz LI, Dijksterhuis A, Holland RW 2017 Training impulsive choices for healthy and sustainable food. J Exp Psychol Appl. 23:204–215. 28150960 10.1037/xap0000112

[ref49] Vlaev I, Chater N, Stewart N, GD a B 2011 Does the brain calculate value? Trends Cogn Sci. 15:546–554. 21983149 10.1016/j.tics.2011.09.008

[ref50] Weber EU, Johnson EJ 2006 Constructing preferences from memory. SSRN Electron J. 1–26.

[ref51] Wimmer GE, Shohamy D 2012 Preference by association: how memory mechanisms in the hippocampus bias decisions. Science. 338:270–273. 23066083 10.1126/science.1223252

[ref52] Wood W, Neal DT 2016 Healthy through habit: interventions for initiating & maintaining health behavior change. Behav Sci Policy. 2:71–83.

[ref53] Worsley K. J. 2001 Statistical analysis of activation images. Functional MRI: an introduction to methods. 14(1), 251–70.

[ref54] Zajonc RB 1968 Attitudinal effects of mere exposure. J Pers Soc Psychol. 9:1–27.5667435

[ref55] Zoltak MJ, Veling H, Chen Z, Holland RW 2017 Attention! Can choices for low value food over high value food be trained? Appetite. 124:124–132. 28627402 10.1016/j.appet.2017.06.010

