# Supplementary material for: Enhanced Bottom-Up and Reduced Top-Down fMRI Activity Is Related to Long-Lasting Nonreinforced Behavioral Change
Source: Cereb Cortex. 2019 Aug 13;30(3):858–74. doi: 10.1093/cercor/bhz132 (PMC7132905; doi:10.1093/cercor/bhz132)
Supplement: BotvinikNezer_CerebralCortex_final_submission_supp_bhz132 [file botviniknezer_cerebralcortex_final_submission_supp_bhz132.pdf]

Enhanced bottom-up and reduced top-down fMRI activity is related to long-lasting non-reinforced behavioral change

Short title: Bottom-up driven long-lasting behavioral change

Rotem Botvinik-Nezer[1,2], Tom Salomon[2] and Tom Schonberg[1,2*]

[1] Sagol School of Neuroscience, Tel Aviv University, Tel Aviv, Israel.
[2] Faculty of Life Sciences, Department of Neurobiology, Tel Aviv University, Tel Aviv, Israel.
* Corresponding author (schonberg@post.tau.ac.il)

Supplementary Materials

Supplementary analyses

Supplementary analysis: behavioral old / new recognition results

Immediately after CAT: As noted in the methods section, we excluded trials with RTs longer than three standard deviations above the mean, for each version of the task. Overall, 0.025% of trials were excluded from the results of the binary version (with RT longer than 3.211 secs) and 0.022% of trials were excluded from the results of the confidence version (with RT longer than 5.536 secs). There was a prominent ceiling effect in participants' performance on the recognition task with a mean hit rate of 99.29% (SD = 2.08%) and mean correct rejection rate of 94.16% (SD = 5.22%); mean d' = 3.921 (SD = 0.547). The mean RT was 1.467 (SD = 0.1389) seconds for hits, 1.828 (SD = 1.541) seconds for misses, 1.519 (SD = 0.420) seconds for correct rejections and 2.220 (SD = 0.924) seconds for false alarms.

Hit rate in the old / new recognition task was only descriptively higher for Go (mean = 99.77%, SD = 1.39%) compared to NoGo (mean = 99.05%, SD = 3.37%) items (logistic regression, two-sided p = 0.181, z = 1.339). RTs in the recognition task were also only descriptively shorter for Go (mean = 1.419 secs, SD = 0.40 secs) compared to NoGo (mean = 1.453 secs, SD = 0.41 secs) items (linear regression, two-sided p = 0.404, t = - 0.836).

One-month follow-up: It should be noted again that the recognition task in the follow-up session was performed after the passive viewing and probe tasks. Therefore, results reflect within-session memory, and not long-term memory effects from the first session. In the follow-up session, the old / new recognition task was performed with the confidence version (n = 23 participants). 0.023% of trials were excluded from analysis (with RTs longer than 5.088 seconds). There was a prominent ceiling effect in participants' performance in the recognition task ($M_{(Hit)}$ = 97.81%, $SD_{(Hit)}$ = 2.37%; $M_{(Correct\ Rejection)}$ = 94.26%, $SD_{(Correct\ Rejection)}$ = 7.77%; $M_{(d')}$ = 3.79, $SD_{(d')}$ = 0.715). The mean RT was 1.486 (SD = 0.384) seconds for hits, 1.671 (SD = 1.260) seconds for misses, 1.509 (SD = 0.506) seconds for correct rejections and 2.403 (SD = 1.014) seconds for false alarms.

Hit rate in the old / new recognition task was only descriptively higher for Go (mean = 98.16%, SD = 3.58%) compared to NoGo (mean = 97.83%, SD = 3.74%) items (logistic regression, two-sided p = 0.747, z = 0.323). RTs in the recognition task were also only descriptively shorter for Go (mean = 1.450 secs, SD = 0.39 secs) compared to NoGo (mean = 1.461 secs, SD = 0.42 secs) items (linear regression, two-sided p = 0.822, t = - 0.225).

Supplementary analysis: behavioral auction results

Similar to previous results regarding the change in WTP following CAT (Schonberg et al., 2014), we observed a general trend of regression to the mean - i.e. while WTP for high-value items decreased, WTP for low-value items increased.

Immediately after compared to before CAT: WTP for high-value items significantly decreased (mean = -0.548 ILS, p = 0.023, two-sided linear mixed model), while WTP for low-value items significantly increased (mean = 0.652 ILS, $p < 0.001$, two-sided linear mixed model). However, the regression to the mean was not significantly different between Go compared to NoGo items, neither for high-value items (Go items: mean ΔWTP = -0.542 ILS; NoGo items: mean ΔWTP = -0.554 ILS; p = 0.918, two-sided linear mixed model), nor for low-value items (Go items: mean ΔWTP = 0.608 ILS; NoGo items: mean ΔWTP = 0.695 ILS; p = 0.482, two-sided linear mixed model).

One-month follow-up compared to before CAT: Similarly, WTP for high-value items significantly decreased (mean = -1.097 ILS, p = 0.002, two-sided linear mixed model) while WTP for low-value items significantly increased (mean = 0.790 ILS, $p < 0.0001$, two-sided linear mixed model) one month after CAT, but this regression to the mean was not significantly different between Go compared to NoGo items, for high-value (Go items: mean ΔWTP = -1.070 ILS; NoGo items: mean ΔWTP = -1.124 ILS; p = 0.688, two-sided linear mixed model) nor for low-value items (Go items: mean ΔWTP = 0.737 ILS; NoGo items: mean ΔWTP = 0.843 ILS; p = 0.453, two-sided linear mixed model).

One-month follow-up compared to immediately after CAT: WTP for high-value items significantly decreased (mean = -0.565 ILS, $p < 0.001$, two-sided linear mixed model) while WTP for low-value items significantly increased (mean = 0.107, $p < 0.0001$, two-sided linear mixed model). Again, the regression to the mean was not significantly different between Go and NoGo items, neither for high-value (Go items: mean ΔWTP = -0.496 ILS; NoGo items: mean ΔWTP = -0.633 ILS; p = 0.439, two-sided linear mixed model) nor for low-value items (Go items: mean ΔWTP = 0.124 ILS; NoGo items: mean ΔWTP = 0.090 ILS; p = 0.805; two-sided linear mixed model).

Supplementary exploratory analyses

Probe

Correlation between choices across sessions. We performed a repeated-measures logistic regression, modelling each follow-up probe choice by the choices made by the same participant in the same probe pair in the first session (each choice could have been made zero, one or two times in the probe

of the first session, since each probe pair was presented twice in each session, once in each block). There was a significant correlation between choices immediately following CAT and choices in the one-month follow-up (proportion of Go item choices in each specific follow-up probe pair as a function of choices of this item in the same specific probe pair in the first session: 0.838 when it was chosen in both blocks in the first session; 0.496 when it was chosen only in one block in the first session; 0.234 when it was not chosen at all in the first session; odds ratio = 17.195; $p < 0.001$).

The effect of CAT on choices above and beyond baseline WTP difference. To test the effect of CAT above and beyond the pre-training WTP, we added the difference in baseline WTP between the two choice alternatives (WTP Go item minus WTP NoGo item) to our logistic model of choosing Go items. WTP difference was significantly related to choosing the Go item (first session, high-value choices, $p < 0.001$, one-sided logistic regression). Importantly, high-value Go items were still significantly chosen over high-value NoGo items, above and beyond the WTP difference ($p = 0.003$, one-sided logistic regression). This was also true for the low-value category (WTP difference $p < 0.001$, choosing Go items $p = 0.041$) and for the follow-up session (high-value items: WTP difference $p < 0.001$, choosing Go items $p = 0.041$; low-value items: WTP difference $p < 0.001$, choosing Go items $p = 0.021$).

Choices response times. For each session, we computed the mean and SD of choices RT across participants, as well as the RTs for choices of Go items and for choices of NoGo items separately. We only included Go / NoGo probe trials (and not the "sanity checks"). In addition, we modelled the odds of choosing the Go item based on the choice RT with a logistic regression model. We also compared RTs for choices that were congruent with the initial ranking (i.e. choices of the higher-ranked item) compared to choices that were incongruent with the initial ranking (i.e. choices of the lower-ranked item).

After CAT. On average, participants made their choices after 955.18 ms (SD = 69.62 ms). Choices of Go items were significantly faster than choices of NoGo items (Go: mean = 941.40 ms, SD = 74.36 ms; NoGo: mean = 979.87 ms, SD = 72.69 ms; $p < 0.001$, two-sided logistic regression). Choice RTs were not significantly different between choices of the higher-ranked items and choices of the lower-ranked items, neither when the Go item was chosen (choices of higher-ranked items: mean = 936.53 ms, SD = 74.00 ms; choices of lower-ranked items: mean = 940.66 ms, SD = 89.48 ms; $p = 0.488$, two-sided logistic regression) nor when the NoGo item was chosen (choices of higher-ranked items: mean = 978.86 ms, SD = 84.70 ms; choices of lower-ranked items: mean = 986.15 ms, SD = 70.71 ms; $p = 0.853$, two-sided logistic regression).

One-month follow-up. On average, participants made their choices after 922.07 ms (SD = 75.83 ms). Choices of Go items were significantly faster than choices of NoGo items (Go: mean = 914.88 ms, SD = 69.73 ms; NoGo: mean = 937.76 ms, SD = 87.78 ms; $p < 0.001$, two-sided logistic regression). Choice RTs were not significantly different between choices of the higher-ranked items and choices of the lower-ranked items, neither when the Go item was chosen (choices of higher-ranked items: mean = 911.60 ms, SD = 63.79 ms; choices of lower-ranked items: mean = 917.38 ms, SD = 86.39 ms; $p = 0.405$, two-sided logistic regression) nor when the NoGo item was chosen (choices of higher-ranked items: mean = 928.54 ms, SD = 82.61 ms; choices of lower-ranked items: mean = 942.90 ms, SD = 101.04 ms; $p = 0.116$, two-sided logistic regression).

Auction

Choice effect on WTP. We tested whether there was a choice effect on WTP. We modelled the WTP difference between the auction performed at the end of the first session and the auction performed at the beginning of the first session, by the number of times each item was chosen in the probe task of the first session (performed between these two auctions). In a linear mixed model, we found a significant correlation between the number of times an item was chosen in the first probe and the WTP difference (between the initial auction and the auction performed at the end of the first session, after the probe). While WTP for high-value item decreased in general, WTPs of items that were chosen more in probe showed weaker trend of regression to the mean (p < 0.001). Similarly, WTP of low-value items increased in general, but increased more for items that were chosen more (p < 0.001).

Training

Training response times and choices. Following the procedure of previous CAT studies, each participant had two independent Go-signal delay (GSD) ladders during training: one for high-value Go items and one for low-value Go items. This GSD changed during training according to the success / failure of responses (success = pressing the button before the image disappears from the screen). We tested the correlation between success in training, measured as the mean or final GSD, and the choice effect following CAT. The linear correlation between the mean GSD and the proportion of Go item choices across participants was not significant, neither for high-value items (r = 0.28, $p = 0.099$) nor for low-value items (r = 0.026, $p = 0.880$). The same was true for the final GSD (high-value: r = 0.24, $p = 0.161$; low-value: r = -0.03, $p = 0.858$).

We also tested whether response times during training with respect to the cue onset were related to the proportion of Go item choices across participants. This correlation was not significant, neither for high-value items (r = -0.139, p = 0.450) nor for low-value items (r = 0.116, p = 0.501). Future studies might aim to create a new manipulation to test this.

Eye-tracking

Eye-tracking data were recorded from a subset of participants, using an EyeLink 1000 Plus SR-Research eye-tracker. For the passive viewing task, we had useable eye-gaze data from 10 participants after CAT and 10 participants in the one-month follow-up (we did not obtain eye-tracking data prior to training). Eye-tracking data from the passive viewing task were used to test whether the duration of time spent observing Go items was different from the duration observing NoGo items after CAT. We averaged the time spent looking on Go items and the time spent looking on NoGo items for each participant and performed a paired t-test to test for differences in observation time between Go and NoGo items.

We found no differences in eye-gaze duration between high-value Go and NoGo items during the task, neither after CAT (mean percent of viewing time: high-value Go 75.3%, high-value NoGo 77.4%; p = 0.512) nor in the follow-up session (mean percent of viewing time: high-value Go 77.9%, high-value NoGo 74.2%; p = 0.266).

Supplementary figures

a.			b.		
**Sorted Bids (ILS)**	**Item**			**pairs**	
			High Go		High No-Go
10	1		7		8
9.2	.		10		9
8.9	.		12	X	11
.	7		13		14
.	.	High-value Items 7:18	15		16
.	.		18		17
.	18				
.	.		Low Go		Low No-Go
.	.		44		43
.	.		45		46
.	.		47		48
.	43		50	X	49
.	.	Low-value Items 43:54	52		51
.	.		53		54
.	54				
0.8	.				
0.4	.				
0.1	60				

Supplementary Figure 1. Diagram of the item selection procedure used in this study.

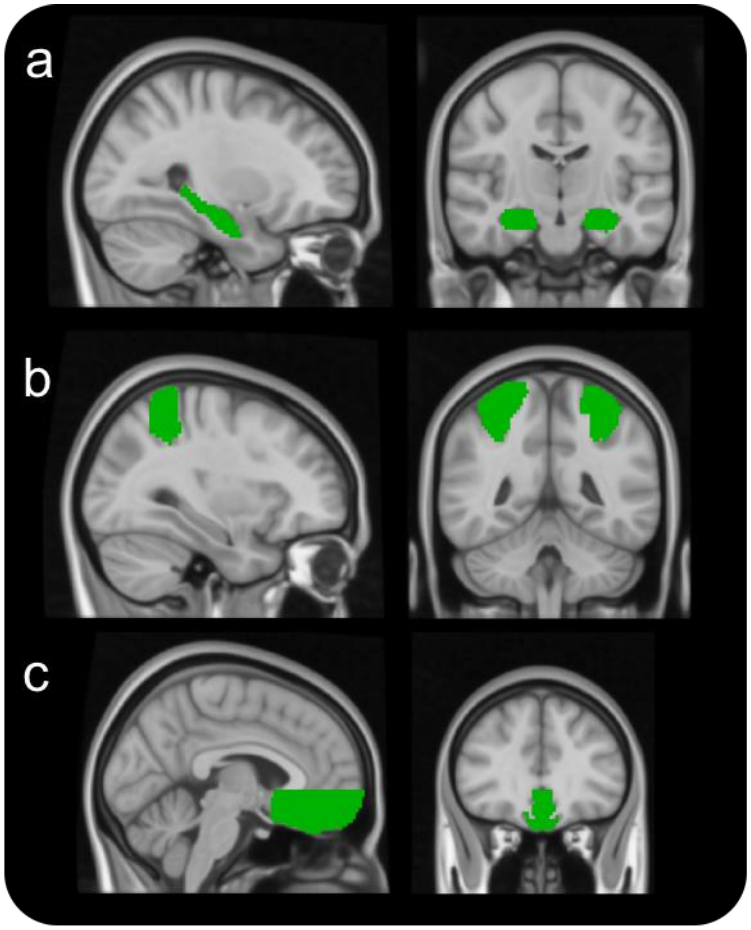

Supplementary Figure 2. Masks for small volume correction: (a) *Left and right hippocampus;* ***(b)*** *left and right SPL;* ***(c)*** *vmPFC.*

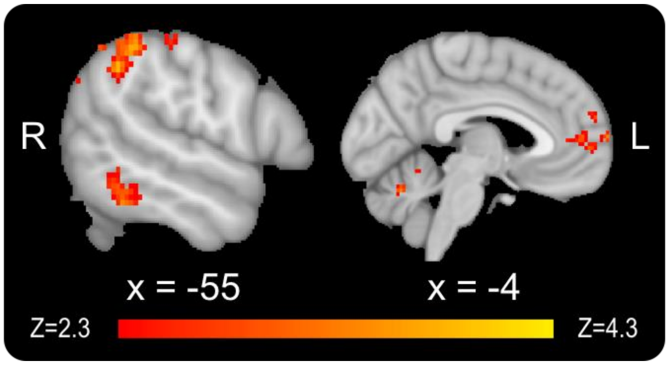

Supplementary Figure 3. fMRI results from the passive viewing task after compared to before CAT, low-value items: *Enhanced BOLD activity in the temporo-occipital part of the left middle temporal gyrus, left superior lateral occipital cortex, left posterior supramarginal / angular gyrus, middle PFC and cerebellum, while passively observing low-value Go compared to low-value NoGo items (whole-brain analysis). For description of all activations see Supplementary Table 4.*

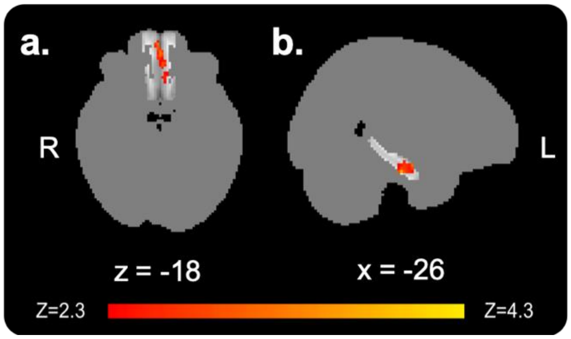

***Supplementary Figure 4. fMRI results from the passive viewing task in the follow-up compared to before CAT, NoGo items:* (a)** *Enhanced BOLD activity in the vmPFC in response to high-value NoGo items (small volume correction).* **(b)** *BOLD activity in response to high-value NoGo items in the left anterior hippocampus was positively modulated by the choice effect across items (small volume correction). The masks used to correct for multiple comparisons in the small volume correction (SVC) analyses are presented on a dark grey brain silhouette. For description of all activations see Supplementary Table 6.*

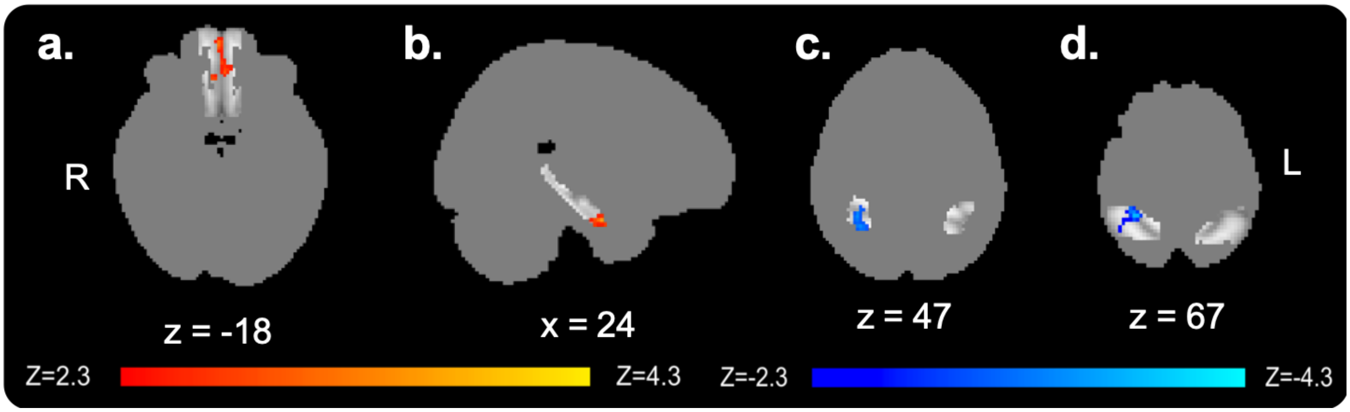

Supplementary Figure 5. fMRI results from the SVC analyses that do not survive Bonferroni correction:* *(a) *Enhanced BOLD activity during passive viewing of high-value Go items one month after CAT compared to before CAT in the vmPFC.* ***(b)*** *BOLD activity during passive viewing of high-value Go items one month after CAT compared to before CAT in the right anterior hippocampus was positively modulated by the choice effect across items.* ***(c)*** *BOLD response was negatively correlated with the choice effect across participants and* ***(d)*** *negatively modulated by the choice effect across items, during choices of high-value Go compared to high-value NoGo items immediately after CAT, in the right SPL.*

The masks used to correct for multiple comparisons in the small volume correction (SVC) analyses are presented on a dark grey brain silhouette. For description of all activations see Supplementary Table 7.

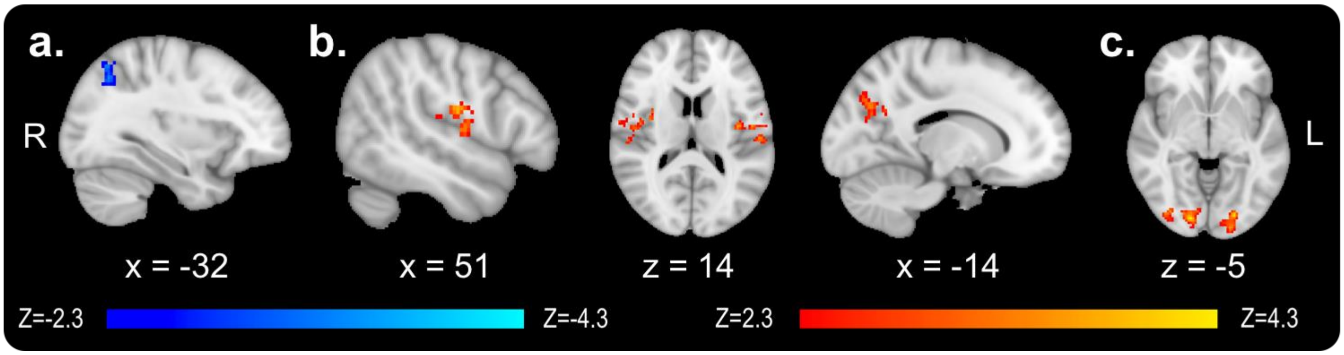

Supplementary Figure 6. fMRI results from the probe task, immediately after and 30 days following CAT, for low-value items and for high-value compared to low-value items: (a) *BOLD activity after CAT was negatively correlated with the choice effect across participants during choices of low-value Go over low-value NoGo items in the left superior lateral occipital cortex (whole-brain analysis).* ***(b)*** *BOLD activity after CAT was stronger during choices of high-value Go items compared to choices of low-value Go items in the left and right Heschl's gyrus / central opercular cortex, as well as in the left precuneous.* ***(c)*** *BOLD activity 30 days after CAT was stronger during choices of high-value Go items compared to choices of low-value Go items in the left and right occipital poles. For description of all activations see Supplementary Table 9.*

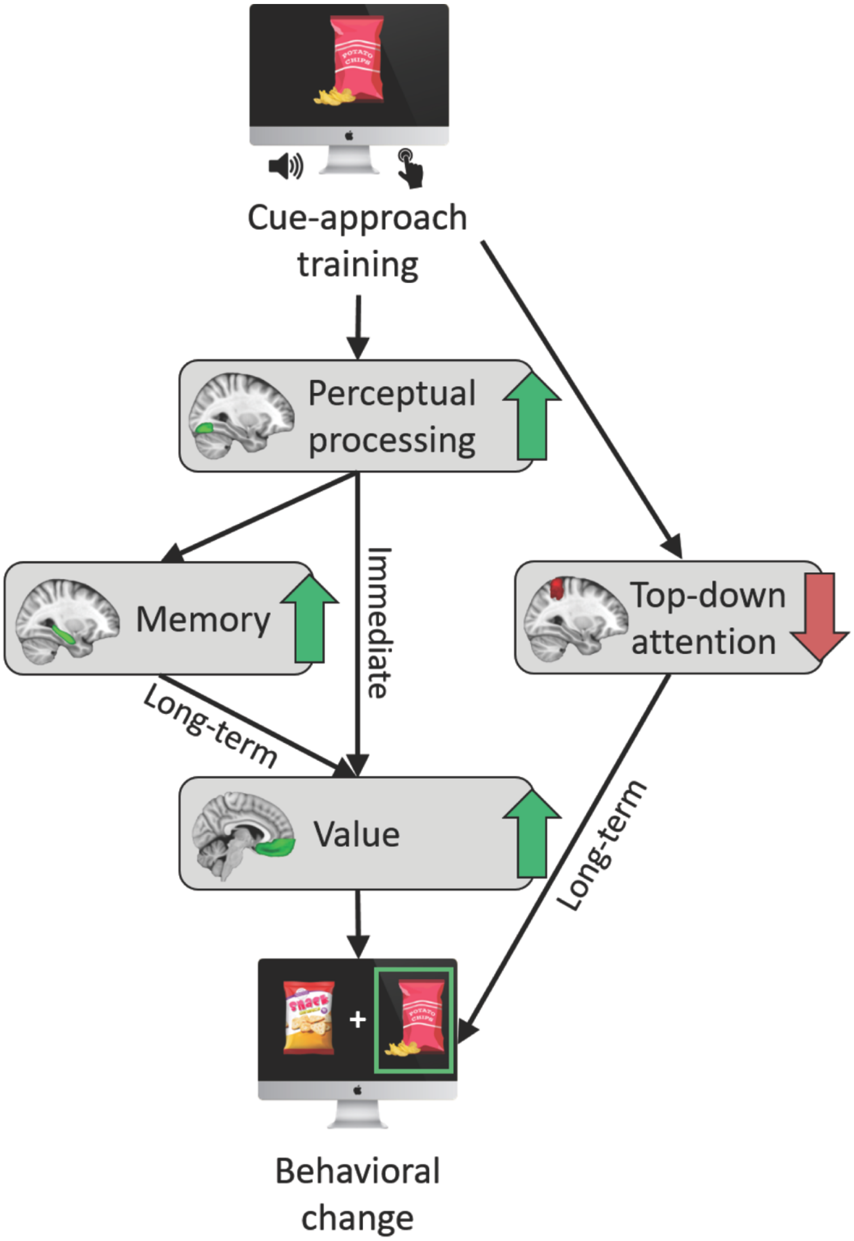

Supplementary Figure 7. Suggested dynamics of the preference change. *We propose that training leads to enhanced perceptual processing, which leads to value enhancement in the short-term and thus to immediate behavioral change. The enhanced perceptual processing putatively enhances memory activation and accessibility, which drives the long-lasting behavioral change. In addition, the involvement of top-down attention is reduced following training, further enhancing the long-term behavioral change. These dynamics are one interpretation of the data that could not be directly tested in the current study and should be tested in future studies.*

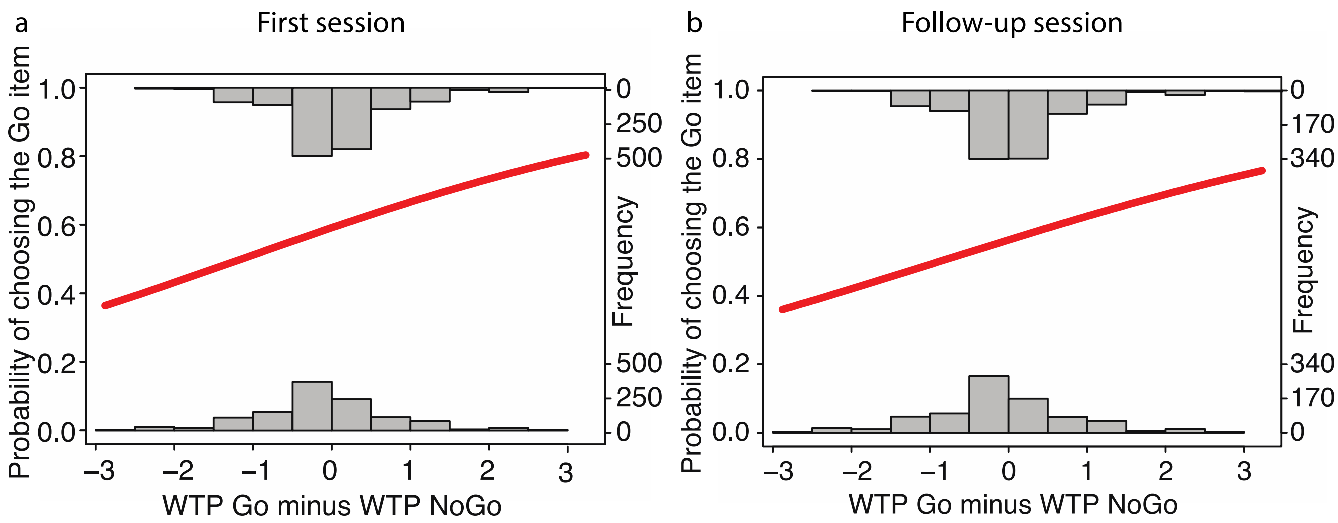

***Supplementary Figure 8. Choice curves reflecting the probability of choosing the high-value Go item over the high-value NoGo item as a function of WTP difference**, in (a) the first session and (b) the follow-up session. The histograms represent the distribution of WTP difference (WTP Go minus WTP NoGo) for choices of Go items (top) and choices of NoGo items (bottom).*

Supplementary tables

	Brand	Description		Brand	Description
**1**	Apropo	Salty snack	**31**	GumiWine	Gummy candy
**2**	BabyDoll	Candy	**32**	Halva	Halva
**3**	BagaleShtuhim	Salty snack	**33**	HappyHippo	Chocolate
**4**	Bamba	Salty snack	**34**	HispusimShatiah	Gummy candy
**5**	BambaNugat	Sweet and salty snack	**35**	Hit	Cookies
**6**	BambaSweet	Sweet and salty snack	**36**	KashitSour	Gummy candy
**7**	BisliBbq	Salty snack	**37**	Keifli	Salty snack
**8**	BisliGrill	Salty snack	**38**	KifkefMaklot	Chocolate
**9**	BisliOnion	Salty snack	**39**	KinderBuenoBrown	Chocolate
**10**	BisliPizza	Salty snack	**40**	KinderBuenoWhite	Chocolate
**11**	Bounty	Chocolate	**41**	KinderJoy	Chocolate
**12**	Cheetos	Salty snack	**42**	Kitkat	Chocolate
**13**	ChocolateParaCookies	Chocolate	**43**	Loacker	Wafer
**14**	ChocolateParaMarir	Chocolate	**44**	Mars	Chocolate
**15**	ChocolateParaMilk	Chocolate	**45**	Mekupelet	Chocolate
**16**	ChocolateParaSucariot	Chocolate	**46**	Mentos	Candy
**17**	ClickBalls	Chocolate	**47**	PesekZman	Chocolate
**18**	ClickBisquit	Chocolate	**48**	Popco	Sweet and salty snack
**19**	ClickBlackWhite	Chocolate	**49**	Shugi	Energy bar
**20**	ClickXLbrown	Chocolate	**50**	SkittlesFruits	Candy
**21**	ClickXLwhite	Chocolate	**51**	SkittlesSour	Candy
**22**	CrunchBisquitBrown	Chocolate	**52**	Smarties	Candy
**23**	CrunchBisquitWhite	Chocolate	**53**	Snickers	Chocolate
**24**	CrunchShokoVanil	Chocolate	**54**	Taami	Chocolate
**25**	DoritosGrill	Salty snack	**55**	Tapuchips	Salty snack
**26**	DoritosNatural	Salty snack	**56**	Tictac	Candy
**27**	DoritosSourSpicy	Salty snack	**57**	Tortit	Chocolate
**28**	Dubonim	Salty snack	**58**	Twist	Wafer
**29**	Egozi	Chocolate	**59**	Twix	Wafer
**30**	GumiSnakes	Gummy candy	**60**	WerthersOriginal	Candy

Supplementary Table 1. Snack food stimuli used in this study.
The complete images data set is available online:
http://schonberglab.tau.ac.il/resources/snack-food-image-database/

	**Order 1 (odd participants)**	**Order 2 (even participants)**
High-value Go items	7,10,12,13,15,18	8,9,11,14,16,17
High-value NoGo items	8,9,11,14,16,17	7,10,12,13,15,18
Low-value Go items	44,45,47,50,52,53	43,46,48,49,51,54
Low-value NoGo items	43,46,48,49,51,54	44,45,47,50,52,53
NoGo items used for “sanity check” probe trials	5-6,55-56	
NoGo filler items	3-4, 19-22, 39-42, 57-58	
Items used only in the BDM auctions	1-2 ,23-38, 59-60	

Supplementary Table 2. Items allocation.

Rank order of the items (based on subjective WTP values) are shown for each set of items (rank 1 = highest). Item selection was counter-balanced across participants, such that for half of the participants ‘order 1’ was used, while for the other half ‘order 2’ was used. NoGo filler items were included in training to maintain a proportion of 30% Go items, but were not presented in the Go-NoGo comparisons during probe. Half of them were high-value items and half of them were low-value items.

Contrast	Cluster	Region	Number of voxels in region	Cluster size	X	Y	Z	Peak z stat	P
High-value Go minus NoGo (Fig 3a)	1	L Lateral Occipital Cortex, inferior division	103	171	-44	-72	-11	4	0.0279
		L Inferior Temporal Gyrus, temporooccipital part	36						
		L Temporal Occipital Fusiform Cortex	14						
	2	R Temporal Occipital Fusiform Cortex	105	192	30	-46	-8	3.82	0.0137
		R Lingual Gyrus	31						
		R Occipital Fusiform Gyrus	22						
		R Lateral Occipital Cortex, inferior division	20						
High-value Go, small volume correction (vmPFC; Fig 3b)	1	Frontal Medial Cortex	78	98	-10	48	-11	3.44	0.0037

Supplementary Table 3. Passive viewing task, after minus before CAT: *Regions with significant activation for the imaging contrasts of Figure 3. For each cluster, the list presents all regions from the Harvard-Oxford atlas that contained at least 10 active voxels within the cluster, as well as the X/Y/Z location for the peak activation in MNI space.*

Contrast	Cluster	Region	Number of voxels in region	Cluster size	X	Y	Z	Peak z stat	P
Low-value Go minus NoGo (Supp. Fig 3)	1	Cerebellum	122	160	-42	-46	-43	4.16	0.0363
	2	Precentral Gyrus	57	217	-54	12	7	4.05	0.0052
		Inferior Frontal Gyrus pars opercularis	50						
		Central Opercular Cortex	34						
		Temporal Pole	17						
		Planum Polare	16						
	3	Inferior Frontal Gyrus pars opercularis	91	237	44	20	-10.5	3.45	0.0027
		Temporal Pole	33						
		Frontal Orbital Cortex	28						
		Inferior Frontal Gyrus pars triangularis	15						
	4	Lateral Occipital Cortex superior division	190	267	22	-72	54.5	3.74	0.0011
		Precuneous Cortex	68						
	5	Insular Cortex	113	297	34	18	9.5	4.2	0.0004
		Frontal Operculum Cortex	104						
	6	Supramarginal Gyrus posterior division	99	326	-58	-54	44.5	4.02	0.0002
		Supramarginal Gyrus anterior division	75						
		Postcentral Gyrus	72						
		Angular Gyrus	16						
	7	Precentral Gyrus	160	435	-30	-6	47	4.66	<0.0001
		Superior Frontal Gyrus	154						

	Middle Frontal Gyrus	62						
8	Juxtapositional Lobule Cortex (formerly Supplementary Motor Cortex)	180	436	8	8	54.5	4.36	<0.0001
	Paracingulate Gyrus	150						
	Superior Frontal Gyrus	44						
	Cingulate Gyrus anterior division	19						
9	Supramarginal Gyrus posterior division	190	505	40	-56	64.5	3.67	<0.0001
	Superior Parietal Lobule	146						
	Postcentral Gyrus	40						
	Angular Gyrus	37						
	Lateral Occipital Cortex superior division	17						
10	Middle Frontal Gyrus	198	540	34	0	64.5	4.59	<0.0001
	Precentral Gyrus	141						
	Superior Frontal Gyrus	138						

Supplementary Table 4*. *Passive viewing task, after minus before CAT, low-value items: *Regions with significant activation for the imaging contrasts of Supplementary Figure 3. For each cluster, the list presents all regions from the Harvard-Oxford atlas that contained at least 10 active voxels within the cluster, as well as the X/Y/Z location for the peak activation in MNI space.*

Contrast	Cluster	Region	Number of voxels in region	Cluster size	X	Y	Z	Peak z stat	P
High-value Go, modulation across items (Fig 4a)	1	L Frontal Pole	139	147	-28	46	-15.5	3.59	0.0203
High-value Go minus NoGo, negative correlation across participants, small volume correction (SPL; Fig 4b)	1	R Superior Parietal Lobule	86	87	30	-40	44.5	3.92	0.0094

Supplementary Table 5*. *Passive viewing task, one month after minus before CAT: *Regions with significant activation for the imaging contrasts of Figure 4. For each cluster, the list presents all regions from the Harvard-Oxford atlas that contained at least 10 active voxels within the cluster, as well as the X/Y/Z location for the peak activation in MNI space.*

Contrast	Cluster	Region	Number of voxels in region	Cluster size	X	Y	Z	Peak z stat	P
High-value NoGo, small volume correction (vmPFC; Supp Fig 4a)	1	Frontal Medial Cortex	75	140	0	48	-15.5	3.741	0.0004
		Subcallosal Cortex	45						
		Frontal Pole	19						
High-value NoGo, modulation across items, small volume correction (hippocampus; Supp Fig 4b)	1	Left Hippocamp us	61	62	-26	-18	23	3.67	0.0109

Supplementary Table 6*. *Passive viewing task, one month after minus before CAT, NoGo items: *Regions with significant activation for the imaging contrasts of Supplementary Figure 4. For each cluster, the list presents all regions from the Harvard-Oxford atlas that contained at least 10 active voxels within the cluster, as well as the X/Y/Z location for the peak activation in MNI space.*

Contrast	Cluster	Region	Number of voxels in region	Cluster size	X	Y	Z	Peak z stat	P
High-value Go, small volume correction (vmPFC; Supp. Fig 5a)	1	Frontal Medial Cortex	47	58	2	56	-18	3.21	0.0227
		Frontal Pole	11						
High-value Go, modulation across items, small volume correction (hippocampus; Supp. Fig 5b)	1	Right Hippocampus	32	36	26	-6	-25.5	3.79	0.0447
Choices of high-value Go items, negative correlation across participants, small volume correction (SPL; Supp. Fig 5c)	1	R Superior Parietal Lobule	52	53	34	-48	47	3.44	0.0345
Choices of high-value Go items, negative modulation across items, small volume correction (SPL; Supp. Fig 5d)	1	R Superior Parietal Lobule	47	47	26	-44	67	3.8	0.0476

***Supplementary Table 7*. SVC uncorrected results:** *Regions with activation that was not significant with a Bonferroni-corrected threshold, for the imaging contrasts of Supplementary Figure 5. For each cluster, the list presents all regions from the Harvard-Oxford atlas that contained at least 10 active voxels within the cluster, as well as the X/Y/Z location for the peak activation in MNI space.*

Contrast	Cluster	Region	Number of voxels in region	Cluster size	X	Y	Z	Peak z stat	P
Choices of high-value Go minus choices of high-value NoGo items (Fig 5a)	1	R Occipital Fusiform Gyrus	63	131	34	-60	-18	3.86	0.0484
		R Temporal Occipital Fusiform Cortex	36						
		R Lingual Gyrus	27						
	2	L Central Opercular Cortex	60	136	-68	-12	12	3.74	0.0391
		L Postcentral Gyrus	10						
	3	R Central Opercular Cortex	94	152	46	-10	7	3.91	0.0200
		R Insular Cortex	14						
		R Heschl's Gyrus (includes H1 and H2)	10						
	4	L Middle Temporal Gyrus, temporooccipital part	64	188	-48	-60	12	4.04	0.0047
		L Lateral Occipital Cortex, inferior division	40						
		L Angular Gyrus	24						
		L Lateral Occipital Cortex, superior division	21						
Choices of high-value Go minus choices of high-value NoGo items,	1	Right Thalamus	104	137	8	-26	2	3.91	0.0375
	2	Left Caudate	65	148	-10	0	7	4	0.0236
		Left Accumbens	11						
	3	R Frontal Operculum Cortex	84	187	30	28	4.5	4.17	0.0049
		R Insular Cortex	29						

negative correlation across participants (Fig 5b)		R Inferior Frontal Gyrus, pars triangularis	15						
		R Frontal Orbital Cortex	11						
	4	L Frontal Operculum Cortex	62	269	-44	22	12	4.51	0.0002
		L Frontal Orbital Cortex	49						
		L Inferior Frontal Gyrus, pars opercularis	34						
		L Insular Cortex	27						
	5	Superior Frontal Gyrus	174	404	2	12	52	4.28	<0.0001
		Juxtapositional Lobule Cortex (formerly Supplementary Motor Cortex)	132						
		Paracingulate Gyrus	34						
Choices of high-value Go items, negative modulation across items (Fig 5c)	1	Superior Frontal Gyrus	104	143	26	8	60	3.98	0.0303
		Middle Frontal Gyrus	19						
	2	L Central Opercular Cortex	66	158	-48	-18	17	3.86	0.0163
		L Parietal Operculum Cortex	59						
		L Planum Temporale	18						
	3	L Frontal Pole	90	225	-40	30	35	4.51	0.0013
		L Middle Frontal Gyrus	88						
	4	R Paracingulate Gyrus	152	242	8	12	27	3.65	0.0007

		R Cingulate Gyrus, anterior division	40						
		R Juxtapositional Lobule Cortex (formerly Supplementary Motor Cortex)	29						
	5	Right Putamen	56	245	32	16	2	3.88	0.0006
		R Insular Cortex	56						
		Right Caudate	25						
		R Frontal Orbital Cortex	20						
		R Frontal Operculum Cortex	13						
	6	L Insular Cortex	63	280	-40	8	9.5	4.43	0.0002
		L Frontal Operculum Cortex	50						
		L Central Opercular Cortex	44						
		Left Putamen	24						
		L Frontal Orbital Cortex	15						
	7	L Precentral Gyrus	164	365	-26	-6	67	4.15	<0.0001
		L Superior Frontal Gyrus	95						
		L Middle Frontal Gyrus	28						

Supplementary Table 8. Probe task, after CAT: *Regions with significant activation for the imaging contrasts of Figure 5a-c. For each cluster, the list presents all regions from the Harvard-Oxford atlas that contained at least 10 active voxels within the cluster, as well as the X/Y/Z location for the peak activation in MNI space.*

Contrast	Cluster	Region	Number of voxels in region	Cluster size	X	Y	Z	Peak z stat	P
Choices of low-value Go minus choices of low-value NoGo items after CAT, negative correlation across participants (Supp. Fig 6a)	1	Lateral Occipital Cortex superior division	148	155	-30	-66	50	3.43	0.0093
Choices of high-value Go items minus choices of low-value Go items after CAT (Supp. Fig 6b)	1	Precuneous Cortex	75	158	-20	-72	17	3.62	0.0159
		Cuneal Cortex	47						
		Lateral Occipital Cortex superior division	24						
	2	Central Opercular Cortex	115	260	-46	-12	20	3.82	0.0003
		Precentral Gyrus	59						
		Postcentral Gyrus	39						
		Planum Temporale	11						
	3	Central Opercular Cortex	157	352	44	-4	17	4.24	<0.0001
		Insular Cortex	20						
Choices of high-value Go items	1	Precuneous Cortex	19	127	-22	-60	9.5	4.16	0.0234

minus choices of low-value Go items one month following CAT (Supp. Fig 6c)	2	Cerebellum	108	238	-38	-72	-25.5	3.86	<0.0001
		Occipital Fusiform Gyrus	19						
	3	Occipital_Pole	140	265	-22	-86	-5.5	3.98	<0.0001
		Occipital Fusiform Gyrus	35						
	4	Occipital Pole	257	588	14	-96	-0.5	4.25	<0.0001
		Lateral Occipital Cortex inferior division	81						
		Lateral Occipital Cortex superior division	58						
		Occipital Fusiform Gyrus	58						
		Lingual Gyrus	31						

Supplementary Table 9. Probe task, immediately after and 30 days following CAT, for low-value items and for high-value compared to low-value items: *Regions with significant activation for the imaging contrasts of Supplementary Figure 6a-c. For each cluster, the list presents all regions from the Harvard-Oxford atlas that contained at least 10 active voxels within the cluster, as well as the X/Y/Z location for the peak activation in MNI space.*

Contrast	Cluster	Region	Number of voxels in region	Cluster size	X	Y	Z	Peak z stat	P
Choices of high-value Go items, modulation across items (Fig 5d)	1	Precuneous Cortex	146	151	6	-70	44.5	3.67	0.0050
	2	R Middle Temporal Gyrus, posterior division	108	175	54	-18	-3	3.89	0.0016
		R Superior Temporal Gyrus, posterior division	24						
		R Superior Temporal Gyrus, anterior division	14						
	3	L Lateral Occipital Cortex, superior division	108	201	-46	-64	24.5	4.18	0.0005
		L Angular Gyrus	61						
	4	R Lateral Occipital Cortex, superior division	205	208	52	-66	34.5	3.87	0.0003
	5	L Middle Temporal Gyrus, posterior division	248	342	-56	-24	-10.5	4.3	<0.0001
		L Superior Temporal Gyrus, posterior division	30						
		L Middle Temporal Gyrus, temporooccipital part	19						
Choices of high-value Go items, correlation across	1	R Postcentral Gyrus	64	181	64	-14	22	4.4	0.0039
		R Parietal Operculum Cortex	41						

participants (Fig 5e)		R Planum Temporale	18						
		R Supramarginal Gyrus, anterior division	18						
	2	Precuneous Cortex	251	474	2	-42	57	3.8	<0.0001
		Cingulate Gyrus, posterior division	183						

Supplementary Table 10. Probe task, one month after CAT: *Regions with significant activation for the imaging contrasts of Figure 5d-e. For each cluster, the list presents all regions from the Harvard-Oxford atlas that contained at least 10 active voxels within the cluster, as well as the X/Y/Z location for the peak activation in MNI space.*
